# Subduction zone fluids and arc magmas conducted by lithospheric deformed regions beneath the central Andes

**DOI:** 10.1038/s41598-021-02430-9

**Published:** 2021-11-29

**Authors:** E. Contreras-Reyes, D. Díaz, J. P. Bello-González, K. Slezak, B. Potin, D. Comte, A. Maksymowicz, J. A. Ruiz, A. Osses, S. Ruiz

**Affiliations:** 1 Departamento de Geofísica, Facultad de Ciencias Físicas y Matemáticas, Universidad de Chile, Blanco Encalada 2002, Santiago, Chile; 2 Centro de Excelencia en Geotermia de Los Andes, CEGA, Santiago, Chile; 3 Departamento de Geología, Facultad de Ciencias Físicas y Matemáticas, Universidad de Chile, Santiago, Chile; 4 Departamento de Ingeniería Matemática, Facultad de Ciencias Físicas y Matemáticas, Universidad de Chile, Santiago, Chile

**Keywords:** Solid Earth sciences, Geophysics

## Abstract

Dehydration of the oceanic subducting slab promotes the formation of magmatic arcs, intra-slab intermediate-depth seismicity, and hydration of the overlying mantle wedge. However, the complex permeability structure of the overriding plate controls the magma and fluid migration and their accumulation at shallower depths. In this regard, mapping the inner structure of the overriding crust and mantle is crucial to understand the magmatic and hydrological processes in subduction zones. We integrate 3-D P-wave, $V_p/V_s$, and electrical resistivity tomographic models of the northern Chilean subduction zone to map the magmatic and fluids derived from the subducting oceanic Nazca plate. Results show a continental crust relatively thick (50–65 km) characterized by a lower zone of high $V_p$ values (7.2–7.6 km/s), which is interpreted as the presence of plutonic rocks. The mantle lithospheric wedge is weakly hydrated ($V_p/V_s$ = 1.75–1.8) while the forearc continental crust is traversed by regions of reduced electrical resistivity values ($< 10^2$ $\Omega m$) interpreted as zones of relatively high permeability/fracturing and fluid content. These regions spatially correlate with upper plate trans-lithospheric deformation zones. Ascending melts accumulate preferentially in the back-arc, whereas hydrothermal systems form trenchward of the volcanic arc. The results highlight the complex permeability structure of the upper South American plate.

## Introduction

The release of fluids in subduction zones is an important process controlling earthquakes both in the subducting/oceanic plate and in the seismogenic megathrust fault zone^1–3^. Dehydration reactions depends on the pressure-temperature conditions of the subducting slab and might trigger mantle wedge serpentinization (20–40 km depth^4,5^), intraplate seismic activity (>60–80 km depth^3,6^), and arc magmatism (> 100 km depth^7,8^). Several geophysical studies suggest that hydration of the oceanic plate is most vigorous at the trench-outer rise prior to subduction, where extensional bending-related faulting affects the hydrogeology of the oceanic crust and mantle^6,9–12^. A larger amount of seawater is more likely to be stored in subduction zones composed by an old and cold oceanic plate that hosts a thicker brittle faulting regime in the upper part of the oceanic lithosphere^10,11^. In fact, old and cold oceanic lithosphere usually present well developed bending-related faults of deep penetration (> 6 km below seafloor) and a large population of horst-and-grabens structures of high vertical offset^6,7,12^. Classical examples include Marianas^13^, Kuril^14^, Tonga^10,15^, and northern Chile^9^.

As the temperature and pressure increases with depth during subduction of a hydrated oceanic plate, metamorphic dehydration reactions take place^5,7,8^. For instance, dewatering of subducting sediments and oceanic crust at depths between 30 and 70 km has been invoked to explain mantle wedge serpentinization^4,5,16,17^. Methamorphic dehydration processes of the subducting oceanic crust increase pore pressure and decrease effective confining pressure, thereby promoting intra-crustal seismicity^1,16^ (60-80 km depth). At depths larger than 100 km, the subducting upper lithospheric mantle dehydrates lowering the melting temperature of the overlying mantle and causing partial melting^1,5,8^. This process leads to the formation of magma that eventually ascends to form an arc of volcanoes and/or accumulates within the overriding plate to form plutonic bodies^18,19^. Depending on the permeability and inner structure of the overriding crust and mantle, the ascending melts would favorably accumulate in deformation zones dominated by a stress regime of tension^20,21^. Hydraulic fracturing can also lead secondary permeability generation by exsolved hydrothermal fluids from the ascent and depressurization of magma^22^.

The Central Volcanic Zone (CVZ) of the Andes is characterized by the subduction of the oceanic Nazca plate beneath the upper South American Plate (SAP) at 14°-28°S. Here, eruptions of caldera scale complexes resulted in immense Late Miocene to Pliocene ignimbrite deposits overlying basaltic and andesitic volcanic rocks, particularly related to the Altiplano-Puna volcanic complex^23^. Today, the active volcanic segment extended between 16° and 28°S and is characterized by an average angle of subduction of 30° of the Nazca plate at 100 km depth^24,25^. whereas north of 14°S and south of 28°S, the active volcanism vanishes, correlating with sub-horizontal subduction (<10°)^24^. Seismic data show that the continental crust under the Andean orogen is extremely thick (60–70 km in average) below the Precordillera and volcanic arc, and it is 60–65 km thick below the Puna/backarc^4,26,27^. The very thick crust below the CVZ is believed to be caused by the combination of long-term crustal shortening and magmatic underplating, with more crustal shortening in the Precordillera and more magmatic underplating below the volcanic arc^28–30^. Regarding the crustal shortening contribution to crustal growth, geochemical evidence shows an increase in crustal thickness during the Cretaceous followed by a delamination episode^31^. Crustal thickness increases again during the last shortening phase occurred during the last 10–22 Myr^31^.

**Figure 1:**
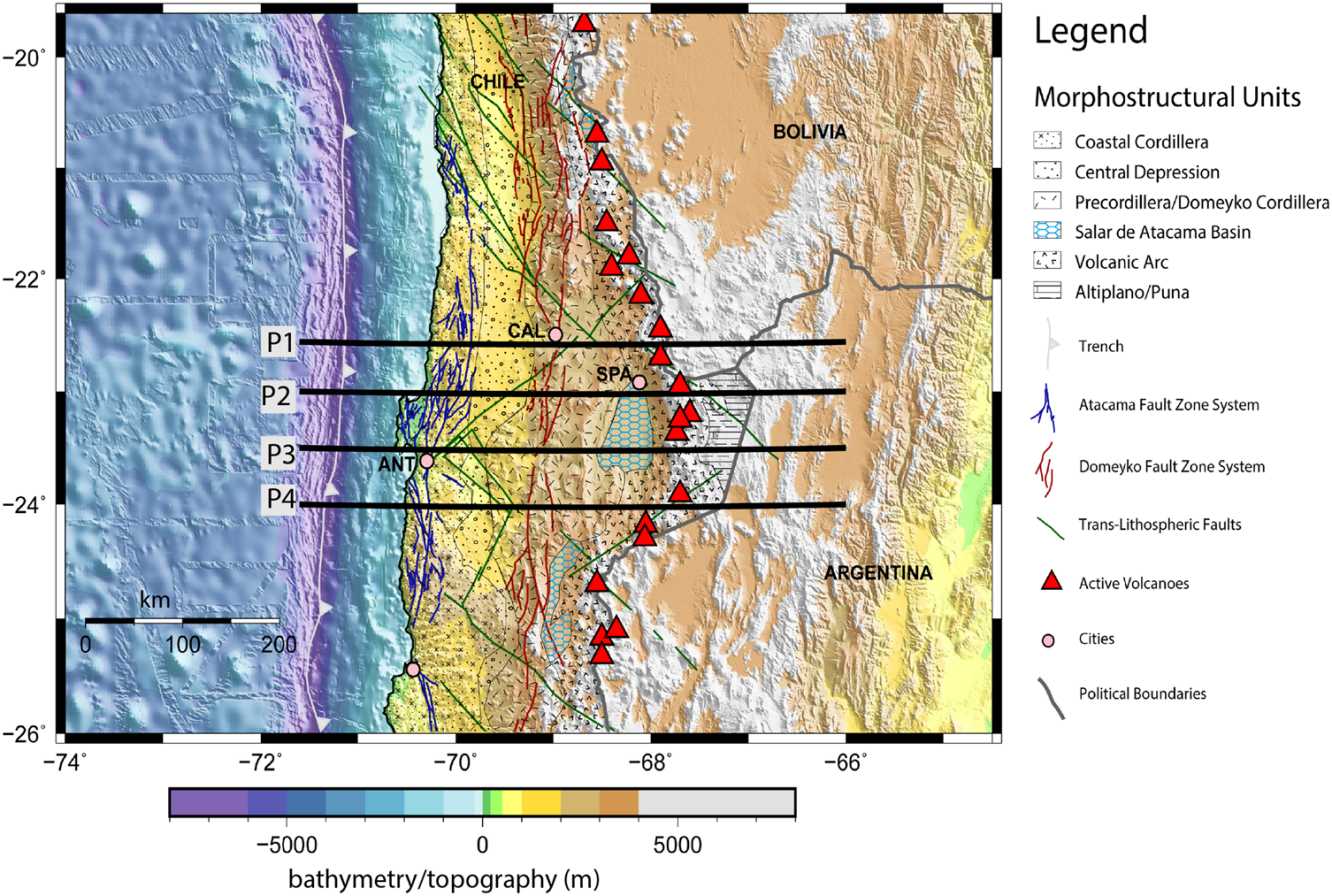
Bathymetric/topographic image of the northern Chilean subduction zone. The black lines denote the extracted profiles for the $V_p$, $V_p/V_s$, and electrical resistivity cross sections shown in Fig. 2. Morpho-tectonic structures are based on the study of Santibañez et al.^38^. ANT: Antofagasta, CAL: Calama, and SPA: San Pedro de Atacama towns. The Atacama and Domeyko Fault Zone Systems (AFZS and DFZS, respectively) aligned approximately parallel to the trench axis. We use the GMT software version 5.0 (https://www.generic-mapping-tools.org) for generating the map.

The western flank of the volcanic arc is limited by the Domeyko Fault Zone System (DFZS; Fig. 1) that was an intra-crustal strike slip fault zone system aligned with the magmatic arc axis during the late Eocene and Early Oligocene^32,33^. Later, the DFZS experienced tectonic inversion evolving from uplift to thrusting facilitating the formation of the Salar de Atacama Basin^34^. On the other hand, the arcward flank of the Coastal Cordillera (a paleomagmatic arc) is delimited by the Atacama Fault Zone System (AFZS), which orientes parallel to the trench axis extending for more than 1100 km along the margin^35^ (Fig. 1).

In this study, we map the structure of the continental crust and mantle wedge in order to explore the presence of fluids and/or magmatic/intrusive bodies, and how they spatially correlate with intra-crustal fault systems (DFZS and AFZS) in the northern Chilean subduction zone. We use a compilation of recently published 3-D P-wave ($V_p$) and P-to S-wave ratios ($V_p/V_s$) models obtained from regional earthquake tomography^25,36^ complemented with 3-D electrical resistivity ($\rho$) models^37^ to study the crustal and upper mantle structure of the SAP beneath the CVZ (Fig. 1). We discuss the processes related to the ascending magma upwellings through a thick and faulted overriding continental crust and their implications for arc volcanism.

## Hydration of the oceanic Nazca plate prior to subduction

Offshore northern Chile, the Nazca plate is 40-50 Myr old, and is characterized by a pronounced fore-bulge or outer rise prior to subduction in the deep trench^10,39,40^. In addition, gravity, active and natural seismic studies show that the oceanic crust and upper mantle of the Nazca plate are pervasively fractured and hydrated at the outer rise region caused by plate bending^3,9,39–41^. Yield strength envelope models predict that the brittle portion for an oceanic lithosphere 40–50 Myr old is 30–40 km thick under tensional stresses, which is about twice thicker than an oceanic lithosphere 10–15 Myr old^39–41^. Thus, the relatively old and cold Nazca plate offshore northern Chile is capable to store a large amount of water when the yield strength of the lithosphere is exceeded and the upper plate is subsequently hydrated. In this regard, flexural models show a systematic reduction in the elastic properties of the Nazca plate likely caused by bending-related faulting in the outer rise region offshore northern Chile^10,39,40^. Thereby, the possibly large amount of seawater stored in the upper Nazca plate has a considerable potential to affect the hydrology, rheology, and magmatism of the overlying SAP via dehydration reactions and partial melting as we discuss in the next sections.

**Figure 2:**
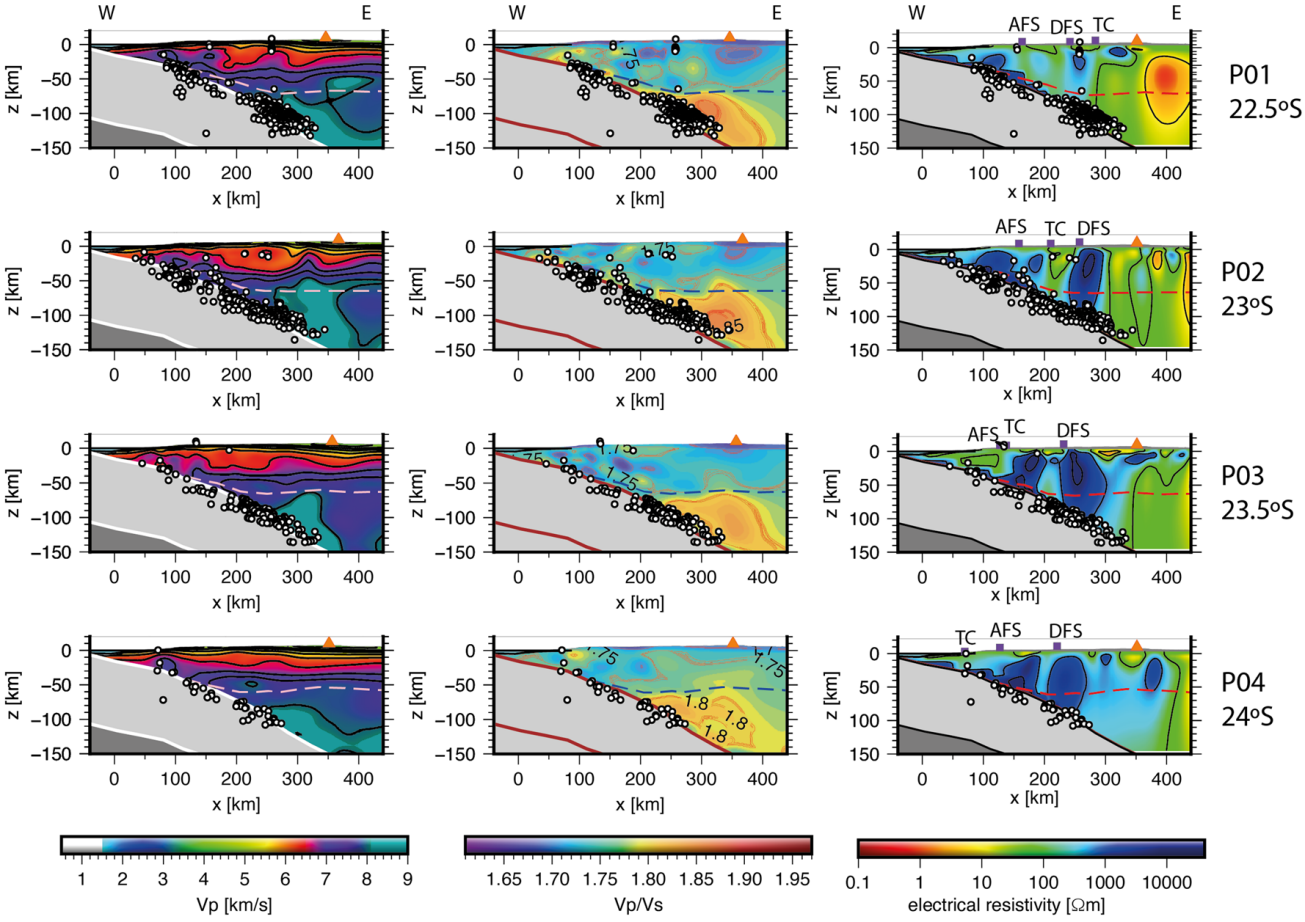
Cross sections for P-wave (left), $V_p/V_s$ (central), and electrical resistivity (right) models along profiles P01–P04 (see Fig. 1 for map location). The dotted curves denote the location of the continental Moho based on the gravity model of Tassara and Echaurren^43^. Seismicity in northern Chile (white dots) is taken from the catalog of Comte et al.^25^. The red triangle indicates the approximate location of the volcanic arc. Tomographic resolution at the edges of the seismic models is decreased due the lack of seismic rays. The shown geophysical models do not constrain the physical properties of the Nazca plate. Nevertheless, we plot the top of the Nazca plate based on the slab 2.0 model of Hayes^44^ corrected by the hypocentre data of Comte et al.^25^. The base of the Oceanic Lithosphere (OL) is based on the 2-D thermal model of Cabrera et al.^3^. The seismic models ($V_p$ and $V_p/V_s$) provide better constraints (in comparison to the $\rho$ model) in the vertical direction (depth-layered structure) mapping the crustal and upper mantle layers. In contrast, the electrical resistivity model defines better (than the seismic models) the lateral extension of the potential magmatic bodies within the continental crust (see Supporting Information for resolution of the geophysical models). We use the GMT software version 5.0 (https://www.generic-mapping-tools.org) for generating the plots.

## The upper continental South American plate

### Geological setting

The locations of the main morpho-structural units are shown in Fig. 1, which are: (1) The Coastal Cordillera (the paleo-magmatic arc) composed by Palaeozoic intrusive, Jurassic-Triassic intrusive, sedimentary and volcanic rocks, (2) The Central Depression (Longitudinal Valley) composed by volcanic and plutonic rocks that underlies younger forearc basins, (3) The Pre-cordillera (Cordillera de Domeyko) is composed by Mesozoic sediments that are pounded unconformably upon metamorphic basement and plutonic rocks, and (4) The Salar de Atacama Basin (SAB) is part of the Pre-Andean Depression formed in the early Late Cretaceous^45^.

### The Atacama fault zone system

The AFZS is a strike slip structure that has undergone periods of inactivity and reactivation since the early Jurassic and controlled the arc magmatism in that time (the present Coastal Cordillera). In the Coastal Cordillera, the magmatism occurred in three primary pulses: a minor Late Jurassic pulse (185–175 Ma), broad Early Cretaceous pulse from 150–120 Ma, and a younger pulse at 120–105 Ma^46^. Fault scarps and surface ruptures indicate different degrees of reactivation of the AFZS up to present that most likely roots into the subduction zone interface indicating the interplay between the AFZS and the subduction interface^47,48^. Moreover, fault activity is expressed as a group of fault-bend fold scarps whose orientation defines three main domains WNW-ESE, N-S and NNW-SSE^49^. These domains were coevally active during the Neogene, accommodating the greatest amount of deformation during the Miocene, continuing their activity during the Pliocene and Lower Pleistocene. The AFZS morphologies and secondary structures suggest that the mechanisms responsible for the deformation are still active^49^.

### The Domeyko fault zone system

The DFZS is a Cenozoic first order structure characterized by a first pulse of Paleocene-Early Eocene deformation characterized by dextral transpression. A second deformation pulse during the Late Eocene-early Oligocene caused relaxation of convergent stresses that reversed the sense of movement on the DFZS and generated transtensional volumes within the zone of structural intersection. During the Neogene-Quaternary the conjugate sinistral NW-SE and dextral SW-NE faults have been also activated^50^. Neotectonic activity during the Plio-Quaternary period in the western flank of the Domeyko Cordillera has also been observed by Audin et al., (2003)^51^ related to fault scarps that cut through alluvial deposits and are distributed parallel to the DFZS.

**Figure 3:**
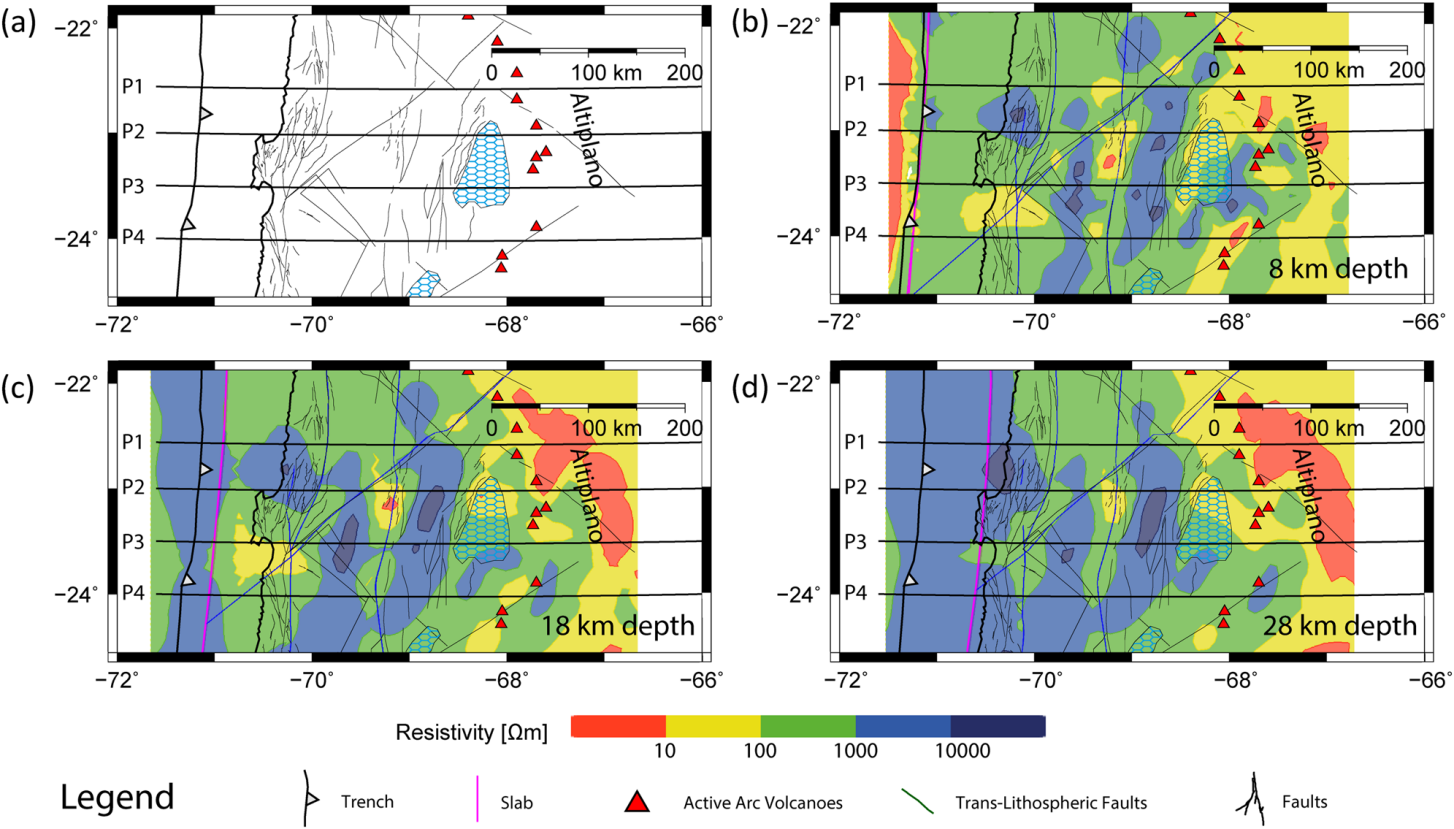
Plan map view for (**a**) major structures in northern Chile between 22°S and 24.5°S from Santibañez et al.^38^. (**b**), (**c**) and (**d**) show the electrical resistivity models^37^ in map view at *z*= 8, 18 and 28 km depth, respectively. The electrical resistivity model is reliable for depths up to 100 km^37^. However, at some regions, there are highly conductive bodies in the upper crust, such as the Salar de Atacama basin, which significantly decreased the penetration depth. Therefore, we show slices for depths shallower than 30 km depth, where the electrical resistivities found in the complete study area (**a**) are reliable according to sensitivity tests (see Supplementary Information). See also Slezak et al.^37^ for more information regarding the electrical resistivity model, including details of the starting model, the grid size/parametrization, the co-variance parameter chosen, error floors employed, the number of iterations, and the final model RMS misfit. We use the GMT version 5.0 (https://www.generic-mapping-tools.org) and Leapfrog Geo version 5.0.1 (https://www.seequent.com) softwares for generating this figure.

## Geophysical results and interpretation

### Seismic constraints

The extracted cross sections for the $V_p$ and $V_p/V_s$ models^36^ are shown in Fig. 2, whereas seismicity was taken from the results of Comte et al., (2016)^25^ who relocate hypocenters using local seismic networks in northern Chile. Most of the seismicity occurred in the oceanic Nazca plate highlighting a high rate of intraplate intermediate depth events in comparison with interplate and crustal seismicity. Similar observations have been reported by other authors^47,52^. In the coastal area, the results show a continental crust 30–40 km thick on average with $V_p$ values of 5.0–6.5 km/s. Similar results were obtained by Contreras-Reyes et al.^41^ using data from a wide-angle seismic experiment that were interpreted as Palaeozoic intrusive and Jurassic-Triassic intrusive rocks.

In the central depression and beneath the volcanic arc, the seismic results show an upper continental crust layer 10–30 km thick with $V_p$ values of 5.0–6.6 km/s that overlies a middle continental crust layer ($V_p$ = 6.6–7.2 km/s) with a variable thickness between 10 and 30 km (Fig. 2). The middle continental crust overlies a seismic layer $\sim$20 km thick (lower crust) with $V_p$ values higher than typical lowermost crust (7.2 km/s) but lower than typical upper mantle (8.1 km/s) defining a High Velocity Zone (HVZ) compared to typical lower crustal $V_p$ values. In addition, the HVZ presents $V_p/V_s$ values of $\sim$1.75 consistent with intermediate composition between mafic and ultramafic rocks^53^. This HVZ was identified in northern Chile by Graeber and Asch^30^, and interpreted as a thick lower crust or partially hydrated uppermost mantle material. Based on the location of the continental Moho^43^ (Figs. 2 and 3), we interpret the HVZ as the lower portion of the continental crust composed of crystallized melts (former Andean magmas) that underplate the crust causing its thickening continuously as the magmatic arc migrates progressively eastward. Eastward migration of the magmatic arc in northern Chile started at least during the Jurassic causing the displacement of the volcanic arc from the Coastal to Western Cordillera^29,54,55^. The eastward migration of the magmatic arc in northern Chile (>200 km) has been explained as the consequence of subduction erosion facilitated by the starved trench due to the hyper-arid climate conditions and subduction of rough seafloor^29,56^.

**Figure 4:**
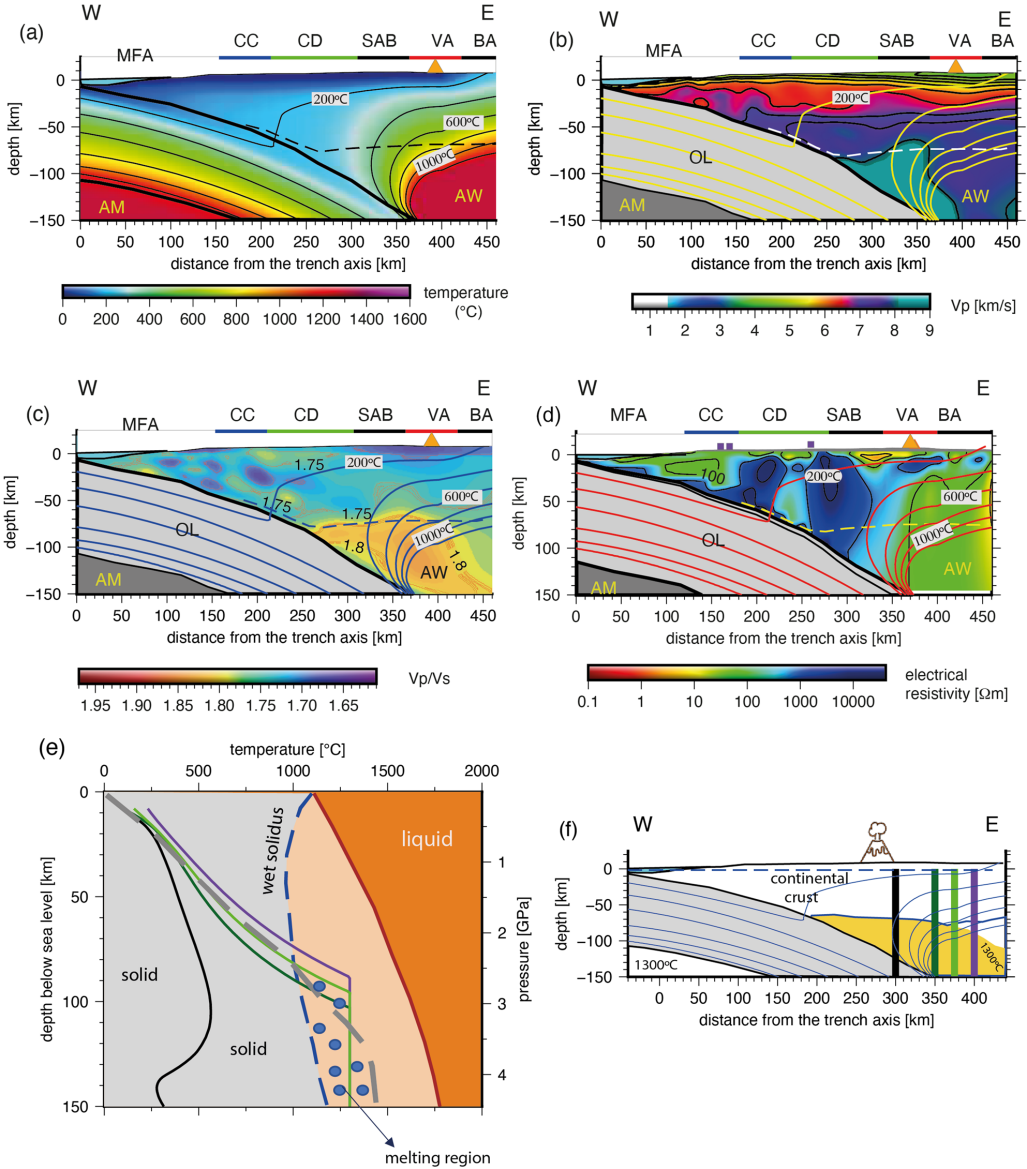
Trench perpendicular geophysical cross section along P03 (23.5°S). (**a**) 2-D thermal model for northern Chile from Cabrera et al.^3^. MF: Marine Forearc, CC: Coastal Cordillera, CD: Central Depression, SAB: Salar de Atacama Basin, VA: Volcanic Arc, BA: Back Arc (Puna), OL: Oceanic Lithosphere, AM; Asthenospheric Mantle, and AW: Asthenospheric Wedge (defined by temperatures higher than 1300 °C). (**b**) $V_p$, (**c**) $V_p/V_s$ ratio, and (**d**) electrical resistivity models. Isotherms are based on the thermal model shown in (**a**). Orange triangle indicates the location of the main active volcano (Lascar and Miñiques). (**e**) Geotherms extracted from the 2-D thermal model shown in (**a**) across *x*=300 (black curve), 350 (dark green curve), 375 (green light curve), and 400 (purple curve) km long-profile as is shown in (**f**). The dashed grey curve corresponds to the average mantle rock geotherm beneath a volcanic arc in subduction zones^8^. The presence of water reduces the solidus temperature (wet-solidus curve) allowing partial melting generation at lower temperatures and lower confining pressures. These physical conditions are reached beneath the volcanic- $\ge$ 90 km depth) and back-arcs ($\ge$ 70 km depth). We use the GMT version 5.0 (https://www.generic-mapping-tools.org) and Inskape version 0.97 (https://www.inskape.org/) softwares for generating the plots.

The location of the continental Moho^43^ (Fig. 2) is coincident with the transition from $V_p/V_s$ values of 1.75 to 1.8 defining the location of the continental mantle wedge. This spatial correlation fails at the eastward edge of the $V_p/V_s$ model likely due to the reduced seismic resolution^36^. The total continental crust thickness in the western magmatic arc is 60-65 km thick, which is consistent with previous seismological studies in the region^4,25,26,30^.

The upper continental mantle velocities are 7.8-8.2 km/s, which is lower than normal mantle rocks (dunite or peridotite; $V_p\sim$8.36 km/s at >1 GPa^53^). According to the laboratory measurements, dry mantle rocks (dunite) at pressures larger than 1 GPa have $V_p/V_s$ values of about 1.76, while 100% hydrated mantle rocks (serpentinite) have $V_p/V_s$ values of approximately 2.1. Thereby, the estimated $V_p/V_s$ values of 1.75-1.8 for the lithospheric mantle wedge corresponds to mantle serpentinization of 10-15%^53^, which are lower than the values reported for Costa Rica ($V_p/V_s \sim$1.85, 15-25% hydration^57^), and for Japan ($V_p/V_s \sim 1.87$, $\sim$30% hydration^58^). A low hydration degree in the lithospheric mantle wedge is also consistent with high electrical resistivity values (>10$^3$ $\Omega m$) determined from a 3-D electrical resistivity model generated from magnetotelluric data acquired in northern Chile^59^. In addition, 3-D body wave seismic tomographic results show a significant contrast in the effects of hydration of the subducted slab on the overriding plate north and south of 21°S^25^. North of this latitude, high $V_p/V_s$ values (1.78-1.8) are attributed to a larger amount of hydration in the subducted Nazca plate that causes the release of larger volumes of water into the mantle wedge. This mechanism has been suggested to influence the nature of volcanism in the magmatic arc and the coupling of the plate boundary at depths of 20-40 km caused by a greater influx of water due to the subduction of oceanic ridges at 20°-22°S^25^.

### Electrical resistivity model

The cross sections extracted from the 3-D electrical resistivity model shows a large zone of reduced $\rho$ (< 10 $\Omega m$) at the back-arc ($\sim$ 400 km landward of the trench axis) along the northernmost profile P01 (Fig. 2). This feature is also present southwards in the back-arc along P02 and P03, but with electrical resistivity values in the range of 1 $< \rho < 10^2$ $\Omega m$^37^.

Along P02-P04, the electrical resistivity model shows zones of variable sizes with high $\rho$ values ($10^3$ $\Omega m$) bounded by pipe-shaped regions with $\rho$ values of $\sim 10^2$ $\Omega m$. $\rho$ values larger than $10^3$ $\Omega m$ fall in the range of igneous and metamorphic rocks^60^, and probably the regions of reduced electrical resistivity ($\sim 10^2$ $\Omega m$) are related to continental rocks with higher hydration and/or metamorphic degree compared to their surroundings. Thus, we interpret these features as the contrast between blocks of relatively low permeability (higher $\rho$ values) with more permeable/fractured rocks (lower $\rho$ values) associated with trans-lithospheric deformation zones. To better illustrate spatially this interpretation, we plot in Fig. 3 the location of the AFZS and DFZS in plan map view with the magnetotelluric model at different depths *z*= 8, 18, and 28 km. In the fore-arc, we observe three N-S elongated low resistivity anomalies that spatially correlate with the trace of the AFZS, DFZS, and below the SAB. A NW trend of small high conductivity zones is also observed in Fig. 3 and is coincident with the Tuina-Caspana trans-lithospheric fault proposed by Yàñez and Rivera-Herrera (2019)^61^. Trans-lithospheric deformed zones have been reported by Vauchez and Tommasi (2003)^62^ and Vauchez et al., (2012)^63^ in Madagascar, Brazil, and Himalayas among other cases. These authors compile several geophysical models to show a coherent deformation of the entire continental lithosphere crosscutting the continental Moho and deforming the upper mantle.

Relatively high electrical resistivity is also observed beneath the volcanic arc at mid- to lower crustal depth (along P03 and P04; Figs. 2 and 3), while a large region of low $\rho$ values is observed at the eastern side of the Western Cordillera in the Altiplano morpho-structural unit. These deep and conductive features have been observed in this area in previous studies between 18° and 23.4°S^59,64–68^ being identified as the Altiplano conductor related to a large volume of inferred partial melt called the Altiplano Puna Magma Body (APMB). The APMB has also been inferred from seismic and gravity studies^69–71^. High conductivity zones below the trace of AFZS and DFZS were already recognized by magnetotelluric studies at 21°S^59,64,72^. Similarly, in the Pre-Andean Depression, the SAB region is characterized by a zone of reduced $\rho$ values (< 1 $\Omega m$), which may be caused by high conductivity fluids beneath the salt pan cover at shallow depths. In fact, in-situ conductivity measurements in different water springs inside the Salar de Atacama showed values up to 12.9 S/m at temperatures of $\sim$ 25 °C^67^ or equivalent to a $\rho$ value of 0.077 $\Omega m$, which is about four times less resistive than the average $\rho$ value of the seawater (0.3 $\Omega m$).

## Discussion

Petrological models predict that metamorphic dehydration processes of the subducting oceanic crust occur at pressures and temperatures higher than 1.2 GPa and 500 °C, respectively^1,5^. This process leads to the blueschist to eclogite transition that dehydrates most of the water chemically bound in subducting oceanic crust leading to serpentinization of the lithospheric mantle wedge^4,30,57,58^. However, the electrical resistivity model shows a heterogenous distribution of regions with reduced $\rho$ values ($< 10^2$ $\Omega m$; Figs. 2-4) usually associated to more permeable regions. These regions are surrounded by fore-arc blocks of high $\rho$ values ($> 10^3$ $\Omega m$), which are interpreted as relatively rigid rocks within colder areas of the continental wedge (< 300 °C) with reduced presence of aqueous fluids and melts.

Meteoric fluids circulate freely in the upper brittle continental crust and dominate magmatic heat sources^65^. In the lower continental crust (ductile regimen), fluids are sourced from devolatilization reactions^5,73,74^ and are confined to isolated hydraulic domains^75–79^. Such domains would behave as weak inclusions imbedded within adjacent fluid-poor rocks^79,80^ and can be connected to an upper crustal drainage network^65^ creating thus a trans-lithopsheric system of flow. Moreover, the possible absence of a permeability discontinuity or barrier at the brittle/ductile transition zone suggests that fluids stored in high-permeability domains in the lower continental crust can be transmitted to the upper continental crust^77^. In the case of a continental crust that is being thickened and heated (like the Andes case), thermally activated viscous compaction processes allow the expulsion and upward migration of fluids in the lower crust^65,78–80^. In addition, tectonism and streamflow responses to earthquakes demonstrate that dynamic stresses can instantaneously change permeability on a regional scale^81^. Thus, we interpret the low $\rho$ anomalies that traversed the rigid blocks in the fore-arc of relatively high $\rho$ regions as highly fractured and hydrated zones associated to trans lithospheric deformation zones (AFZS and DFZS). Likely, the AFZS and DFZS favor upward fluid channeling where fluids are sourced by the metamorphic reactions of the subducted slab.

**Figure 5:**
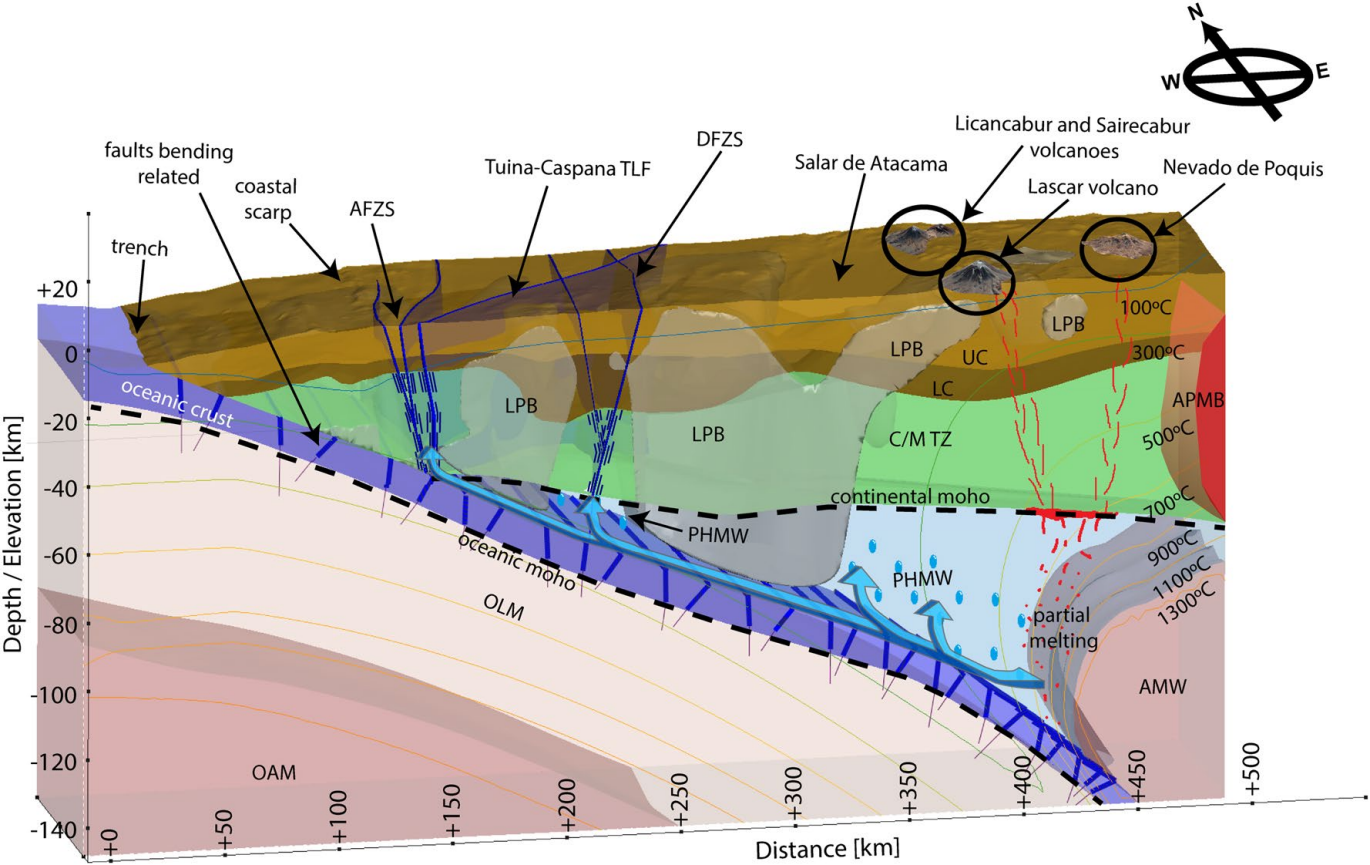
Summarized schematic interpretation of the seismic and magnetotelluric models. AFZS: Atacama Fault Zone System, TLF: Trans Lithospheric Fault, DFZS: Domeyko Fault Zone System, LPB: Low Permeability Block, UC: Upper Crust, LC: Lower Crust, C/MTZ: Crustal/Mantle Transition Zone, PHMW: Partly Hydrated Mantle Wedge, AMW: Asthenospheric Mantle Wedge, OLM: Oceanic Lithospheric Mantle, and OAM: Oceanic Asthenospheric Mantle. The subducting slab dehydration causes mantle wedge serpentinization and provides the flux needed to lower the melting temperature. At depths of 120–150 km, partial melting of ultramafic mantle rocks occurs yielding the production of Andean/mafic magmas. The ascending melts follow a complex trajectory, with upward and arcward migration depending on the permeability structure of the overriding plate^61,82^. Fluids (sourced from the dehydration reactions of the subducted slab) flow trenchward with unimpeded lateral migration along the plate boundary and follow complex upward paths only at the edges of the LPBs^61,82^ (light blue arrows). The western edge of the Altiplano Puna Magmatic Body (APMB) is shown and has been imaged eastwards of the volcanic arc between 18° and 23.4°S by several authors^19,64–68^. We use the GMT version 5.0 (https://www.generic-mapping-tools.org) and Leapfrog Geo version 5.0.1 (https://www.seequent.com) softwares for generating this figure.

The segmentation of the continental mantle wedge in regions of relatively high and low permeability play an important role in the migration of fluids within the upper plate^75,76^ with potential trenchward and arcward paths as has been suggested by numeric models^61,82^. Dehydration reactions from the subducted oceanic crust occurs likely at depths between 30 and <100 km leading to serpentinization of the overriding continental mantle wedge preferentially in regions of higher permeability. This phenomenon has been widely inferred in several subduction zones by seismological studies^25,30,57,58^. The fluids sourced from the subducting slab (below the LPBs) can further flow updip (trenchwards) along the whole plate boundary^61,82^, whereas upward fluid migration is more likely controlled by the location of the LPBs as is illustrated in Fig. 5. For instance, Yañez and Rivera-Herrera (2009)^61^ used a flow model in porous media to show that magmatism tends to migrate laterally until it reaches high permeability paths to the surface. They concluded that the upward fluid migration occurs preferentially at the edges of crustal dense blocks in the northern Chilean forearc where the trans-lithospheric fault zone systems are located.

The electrical resistivity model shows that the LPBs are placed both in the continental crust and mantle wedge allowing the connection of the LPB’s with fluids flowing along the plate boundary (Figs. 2 and 5). Likely, upward fluid migration occurs at the flanks of the LPBs^61,82^, which coincide with the AFZS and DFZS (Fig. 5). Furthermore, upward fluid circulation along the AFZS and DFZS might favor mineralization processes, the formation of hydrothermal systems and magmatic reservoirs^8,64,73^ (Figs. 2 and 5).

Beneath the active volcanic arc and eastward of the larger forearc block (LPB in Fig. 5), $\rho$ values decrease again ($< 10^3$ $\Omega m$). In that zone, at depths >130 km, the thermal model^3^ shows ideal conditions of pressure (> 2.5 GPa) and temperatures (> 500 °C) to produce dehydration reactions from the subducted oceanic mantle causing partial melting and leading to arc volcanism^1,2,8,16^ (Fig. 5). Andean magmas are recognized to have a rich felsic, dacitic and rhyolitic compositions^29^, particularly in the segment characterized by the presence of the Altiplano Puna Volcanic Complex (APVC). The APVC is a major volcano tectonic province developed between 21° and 24°S in response to a late-Miocene ignimbrite flare-up, with a strong volumetric dominance of ignimbrites and related silicic volcanic rocks over more andesitic compositions^23^. The volcanic complex is developed on one of the thickest parts of the Central Andean crust, which seems to be related to the previous large scale silicic volcanism, initiated at about 10 Ma^83^ as a surface expression of silicic magmatism at depth. This previous large-scale volcanism shares a strong physical and petrological resemblance with Late Pleistocene domes as the Chao dacite in Chile^84^ that was found at the western edge of the APVC. However, geophysical and geological evidence from studies carried out in the present days of this segment of the Central Andes suggest the presence of zones of partial melting through the whole crustal column within the APVC, ranging from silicic at shallow levels to mafic compositions at the bottom of the crust^19^.

The results indicate that low-density Andean magmas that buoyantly rises through the heterogenous more permeable regions of the continental plate are subsequently redirected by pressure-temperature and via complex paths as suggested by numeric models^61,82^. For instance, the electrical resistivity model shows a region of relatively high resistivity ($\rho > 10^3$ $\Omega m$) at 10 to 30 km depth south of Lascar volcano (Figs. 4 and 5), which seems to obstruct the ascent of melts in this area. However, this relative high resistivity zone vanishes at $\sim$30 km depth defining a relatively conductive zone which seems to relate to the western border of the Altiplano conductive anomaly^67^. Melts in this area seem to differentiate and accumulate preferably within the APMB as is observed at the western edges along profiles P1-P4 (Figs. 2 and 3). The interaction of ascending magmas with the surrounding continental crustal rocks also leads to partial melting and the formation of hydrothermal systems at shallow levels. For instance, a small-scale but highly conductive region ($\rho<$1 $\Omega m$) can be seen at the trenchward side of the volcanic arc at depths shallower than 10 km, which is coincident with known hydrothermal systems such El Tatio Geysers field, suggesting a possible tectonic control for the crustal emplacement of these features.

The elongated relatively high conductivity zones in the fore-arc that correlate with the AFZS and DFZS can be interpreted as extensive damage/deformed zones caused by successive wall rock failure due to the ascent and subsequent depressurization and volatile saturation during the crystallization of the magma chambers^22,85^. Thus, arc magmatism changes the rheology of the overriding plate by thermally weakening deformed zones^55,86^ that were already created by continuous tectonic stresses associated to the subduction system. The combined effect of arc magmatism and tectonic stresses increase the permeability of the deformed zones favoring fluid migration. In particular, the AFZS and DFZS were coincident with active arc magmatism during the Mesozoic^46^ and Paleocene-Oligocene^87^, respectively. Thus, the interplay between tectonic stresses and former arc magmatism along AFZS and DFZS provide likely the paths for recent redistribution of slab-delivered fluids within the overriding South American plate (Fig. 5).

## Summary

The combined analysis of recently published 3-D $V_p$, $V_p/V_s$, and electrical resistivity models reveal the structure of the overriding South American plate in the northern Chilean subduction zone (22°-24°S) from the trench axis up to the back arc. The results lead to the following conclusions:

1. The continental crust is relatively thick (50-65 km) between the Coastal Cordillera and the back arc. The lower part of the continental crust is characterized by a high velocity zone (7.2-7.6 km/s) defining the lower continental crust composed of plutonic rocks.
2. The continental mantle lithospheric wedge is slightly hydrated with $V_p/V_s$ values of 1.75-1.8 compared to other subduction zones such as Costa Rica and Japan ($V_p/V_s \sim$1.85).
3. The continental crust and mantle wedge are traversed by regions of relatively low electrical resistivity ($< 10^2$ $\Omega m$) compared to their surroundings ($> 10^3$ $\Omega m$). In addition, the shallower parts of the relatively low electrical resistivity regions spatially correlate with the Atacama and Domeyko Fault Zone systems.
4. We interpret the relatively low electrical resistivity regions as zones of higher permeability/fracturing and fluid content associated to the trans-lithospheric deformation zones (Atacama and Domeyko Fault Zone systems). Melt and fluids derived from dehydration reactions from the subducting slab follow complex trench- and arc-ward paths as is suggested by the heterogeneity of the 3-D electrical resistivity model .

## Methods

### Seismological data and modeling

Comte et al.^25^ determined 3D body wave seismic tomography in northern Chile, incorporating surface wave observations to adjust the model before the joint inversion. The data used compromises 342 short-period seismic stations deployed in northern Chile over several decades and 18 broadband stations operated by IPOC, CSN, and ONEMI over three years (1/1/2012–12/31/2014). The data corresponded to a combination of arrival times from earthquake-generated body waves and transit times from ambient noise generated surface waves recorded by seismic stations deployed in northern Chile for different periods over the past three decades. The initial body wave data set derived from these deployments consisted of P and S arrival times from 33,351 events recorded over a period of 25 years by a variety of networks comprising 360 stations. From this data set, earthquakes that were recorded by at least 10 stations and with predicted arrival times within an initial outlier residual threshold of the larger of 2 seconds and 10$\%$ of the total travel time, were selected. Application of these criteria resulted in a reference data set of 11,874 events with 110,640 P and 106,680 S wave arrival times. The simultaneous fitting of body wave arrival times and surface wave transit times was done to determine a 3D body wave seismic tomography. Surface waves were included to reduce potential trade-offs in crustal and mantle structures caused by the lack of local shallow earthquake activity. Surface waves also provided additional constraints on shear wave speeds that compensate for the lower number of, and higher uncertainties in, shear wave arrival times.

The joint inversion methodology was based on an approach described in Roecker et al.^88,89^. The subsurface was then parameterized by specifying P and S velocities on a 3D grid of nodes spaced at 5 km intervals in depth and latitude and about the same distance in longitude. Intragrid velocities were determined by trilinear interpolation. Body wave travel times within the medium were calculated using a 3D Eikonal equation solver in a spherical coordinate system. Hypocenter coordinates were included as variables in the inversion, but they were also relocated in the updated models before any iteration. The models generally needed 10 iterations with only surface wave times followed by an additional 10 iterations with combined surface and body wave times. In this way, Comte et al.^25^ determined that the best way to incorporate surface wave observations was to allow them to adjust the model prior to the joint inversion. For the starting model of the surface wave only inversion, they began with a body wave only inversion that started with an adaptation of the 1D model of Husen et al.^90^ for the Antofagasta region. The resulting 3D body wave model for $V_p$ and $V_p/V_s$, obtained after 13 iterations, was averaged laterally to obtain a 1D estimate of $V_s$, ensuring some compatibility with the body waves and provides a means for determining appropriate values for regularization and thresholds for data misfit. Because surface wave inversions tend to retain biases introduced by interfaces like the Moho, they smoothed the transition from crustal to mantle velocities over a range of depths (40–70 km) suggested by previous estimates of crustal thickness in this area^26,27^. This 1D $V_s$ model was then used as a starting model for the surface wave inversion. After 10 iterations of the surface wave inversion, the velocities in the resulting 3D $V_s$ model were then multiplied by a constant $V_p/V_s$ ratio to construct a starting 3D $V_p$ and $V_s$ model for the joint inversion. Based on both the results from the body wave inversion and a linear fit to $T_p$ vs $T_s$-$T_p$ for the entire body wave data set, a $V_p/V_s$ = 1.76 was obtained as the preferred value. Several trial inversions were also performed to evaluate the effects of a variety of assumptions and choices of parameters on the final model. As a result of the trials, they concluded that the first order features in the preferred model are robust. Specifically, the increase in $V_p/V_s$, from the subducted slab into the supra-slab mantle, as well as the size and location of the high $V_p/V_s$ regions, appeared well constrained. These features are reasonably independent of the starting model, but the actual values of $V_p/V_s$ tend to stay close to the starting value. While large gradients in $V_p$, $V_s$, and $V_p/V_s$ were well constrained, localized regions with small changes (on the order of 1%) are not significant.

### Electrical resistivity model

The magnetotelluric (MT) method is a passive surface geophysical technique that uses the time-varying natural electromagnetic (EM) fields to study the electrical resistivity distribution at depth^91,92^. The relationships between horizontal electric field ($E_h$) and magnetic ($H_h$) fields on the surface

1 $$\begin{aligned} E_h=Z H_h, H_z=T H_h \end{aligned}$$

define the transfer functions *Z* (impedance tensor) and *T* (tipper) that depend on the distribution of electrical resistivity in the subsurface^93^. To obtain a model of the electrical resistivity structure of the lithosphere, a dataset consisting of 58 MT stations at an array of sites distributed between 22° and 24.5°S were used^37^. All measurements were carried out in a geographic coordinate system, with the *x*-axis pointing to the geomagnetic north and the y-axis pointing to the geomagnetic east. For the 3-D inversion, 20 periods equally distributed between 10 and 10,000 s were used. The 3-D electrical resistivity model has been obtained from the inversion of the four components of *Z* and the two *T* components using the ModEM code^94,95^, an EM inverse modeling program based on a standard minimum structure, non-linear conjugate gradients algorithm (NLCG).

## Supplementary Information

Supplementary Information.
