# Supplementary material for: Subduction zone fluids and arc magmas conducted by lithospheric deformed regions beneath the central Andes
Source: Sci Rep. 2021 Nov 29;11:23078. doi: 10.1038/s41598-021-02430-9 (PMC8630066; doi:10.1038/s41598-021-02430-9)
Supplement: Supplementary file 1 — Supplementary Information. [file 41598_2021_2430_MOESM1_ESM.pdf]

Supporting Information

Subduction zone fluids and arc magmas conducted by lithospheric deformed regions beneath the central Andes

E. Contreras-Reyes[1*], D. Díaz[1,2], J.P. Bello-González[3], K. Slezak[1], B. Potin[1], D. Comte[1], A. Maksymowicz[1], J.A. Ruiz[1], A. Osses[4], and S. Ruiz[1]

[1] Departamento de Geofísica, Facultad de Ciencias Físicas y Matemáticas, Universidad de Chile
[2] Centro de Excelencia en Geotermia de Los Andes, CEGA
[3] Departamento de Geología, Facultad de Ciencias Físicas y Matemáticas, Universidad de Chile
[4] Departamento de Ingeniería Matemática, Facultad de Ciencias Físicas y Matemáticas, Universidad de Chile

* Corresponding Author Email: edcontrr@uchile.cl

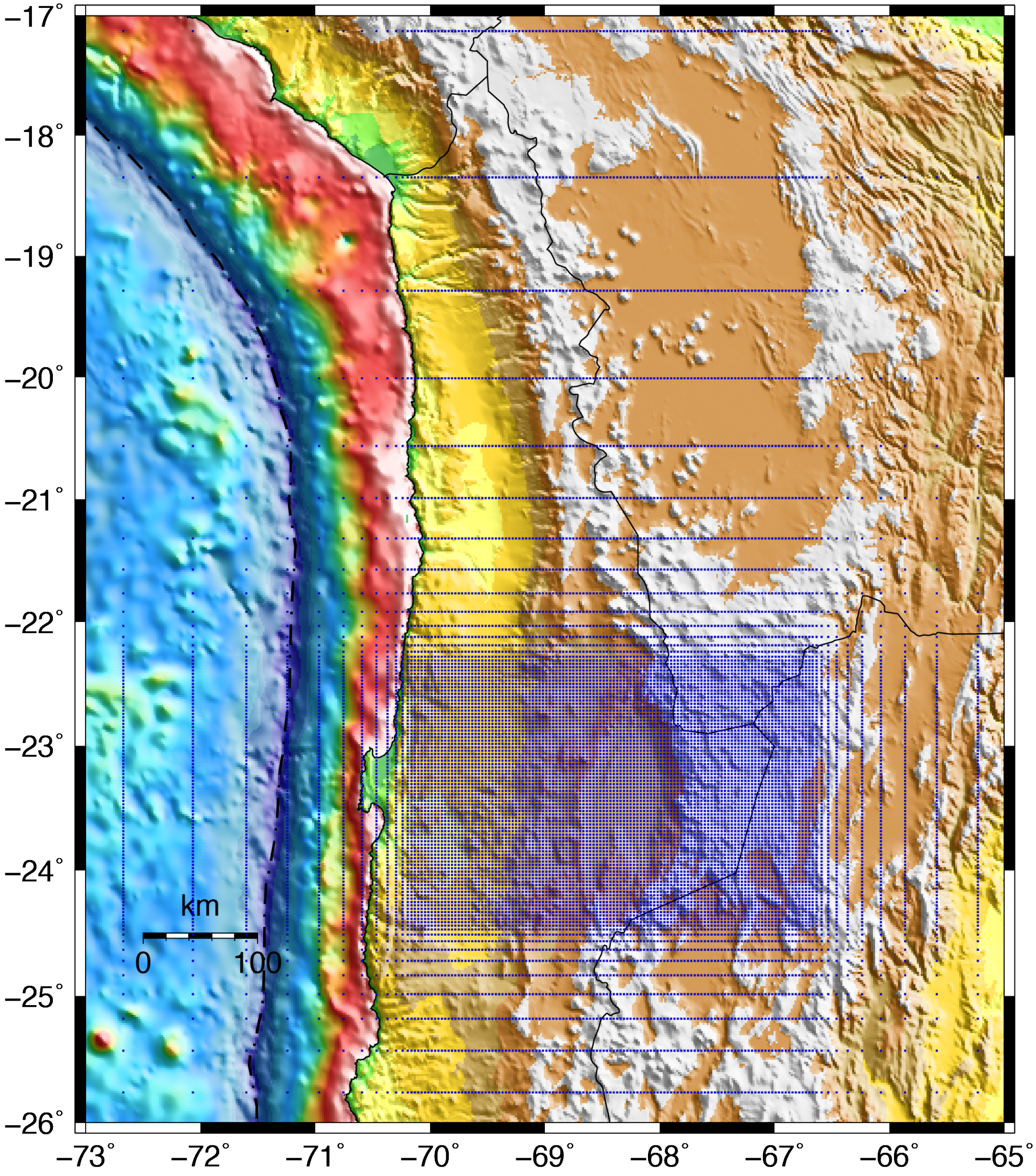

Figure S1. Geometry of the experiment. The blue dots indicate the model-nodes of the electrical resistivity model. We use the GMT software version 5.0 (https://www.generic-mapping-tools.org) for generating the map.

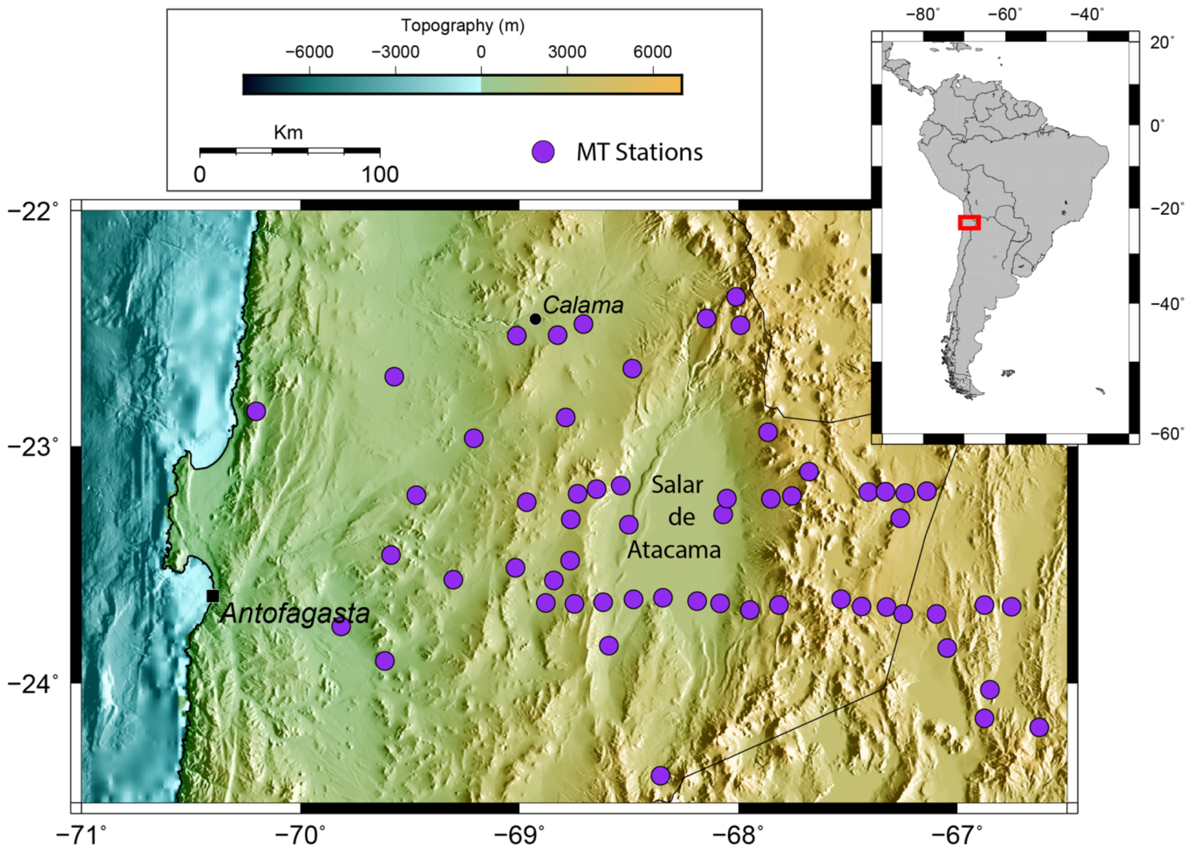

Figure S2.1 Map of the study area with location of long-period magnetotelluric stations (purple dots[1]). We use the GMT software version 5.0 (https://www.generic-mapping-tools.org) for generating the map.

1. Electrical Resistivity Models: Model information and Sensitivity tests

The total grid size used was 112 × 152 × 46, in *x*, *y*, and *z* directions, where the central part of the model held 80 × 120 cells (Fig. S.2.2). The horizontal cell size was 3 km in the central part of the mesh, and then increases by a factor 1.3 with increasing distance from the central part of the model. In the vertical direction the mesh started with 150 m thick cells at the surface and increased by a factor 1.2 with depth.
The initial model was set to a background resistivity of 100 Ωm (a homogeneous half-space) and the bathymetry of the Pacific Ocean was included with the resistivity of the ocean water fixed at 0.3 Ωm.
For the final model the covariance coefficients of 0.3 use used. For the inversion, an error floor of 7% of $|Z_{xy}Z_{yx}|^{1/2}$ was set for all impedance components and the tipper had a fixed

error floor of 0.05. The initial RMS was 18.04 and the inversion converged to a final normalized root mean square (RMS) of 1.55, after 126 iterations.

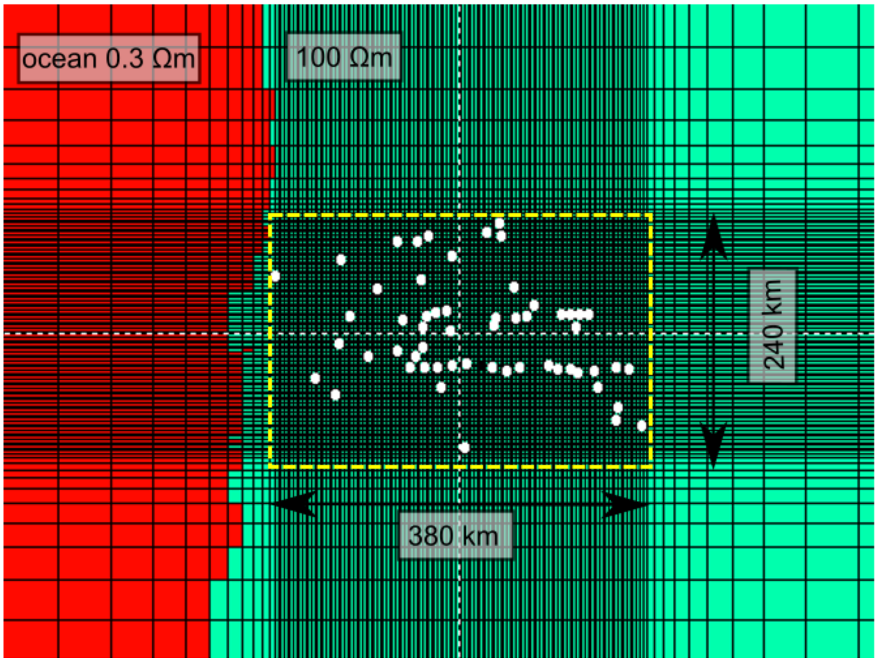

Figure S2.2. The starting model grid at sea level. The yellow dotted line shows the central part of the model (approximately 240 km × 380 km). White dots indicate locations of the magnetotelluric stations. Red color indicates a 0.3-Ωm 3-D body simulating the ocean. We use the GMT software version 5.0 (https://www.generic-mapping-tools.org) for generating the map.

We performed a series of forward modeling tests, replacing each anomaly (C3, C4, C5, C6, C7, and C8) with a resistive, 1000 Ωm body to evaluate whether the data are sensitive to each of these features (see Fig. S1.1 – S2.8 in supplementary material). The tests suggest that all of the anomalies are required to explain the observed data. In all these tests, the overall RMS value always increased after replacement.
For anomaly C2, an additional sensitivity test to check its lateral extension outside the APVC was performed. This structure was replaced by a resistive body, resulting in significant differences in the curves observed at the nearest stations (e.g., sites F05 and F07 in Figure S2.2 in the supplementary material). The test supports that the C2 extension is necessary for the inversion.

In addition, to confirm the vertical extent of C2, C3 and C4, we partially replaced the lower part of these anomalies with resistive blocks. For C2, the top was located at a depth of 58 km, while for C3 it was 23 to 58 km and from 15 to 48 km for C4 (see Fig. S2.3 to Fig. S2.8 in the supplementary material). The sensitivity tests showed that the data require a deep extent of C3, while the depth extent of C4 is poorly resolved by the current set of MT stations, with relatively small changes in the data fit.

1.1 Sensitivity tests - anomalies C3, C4, C5, C6, C7, and C8.

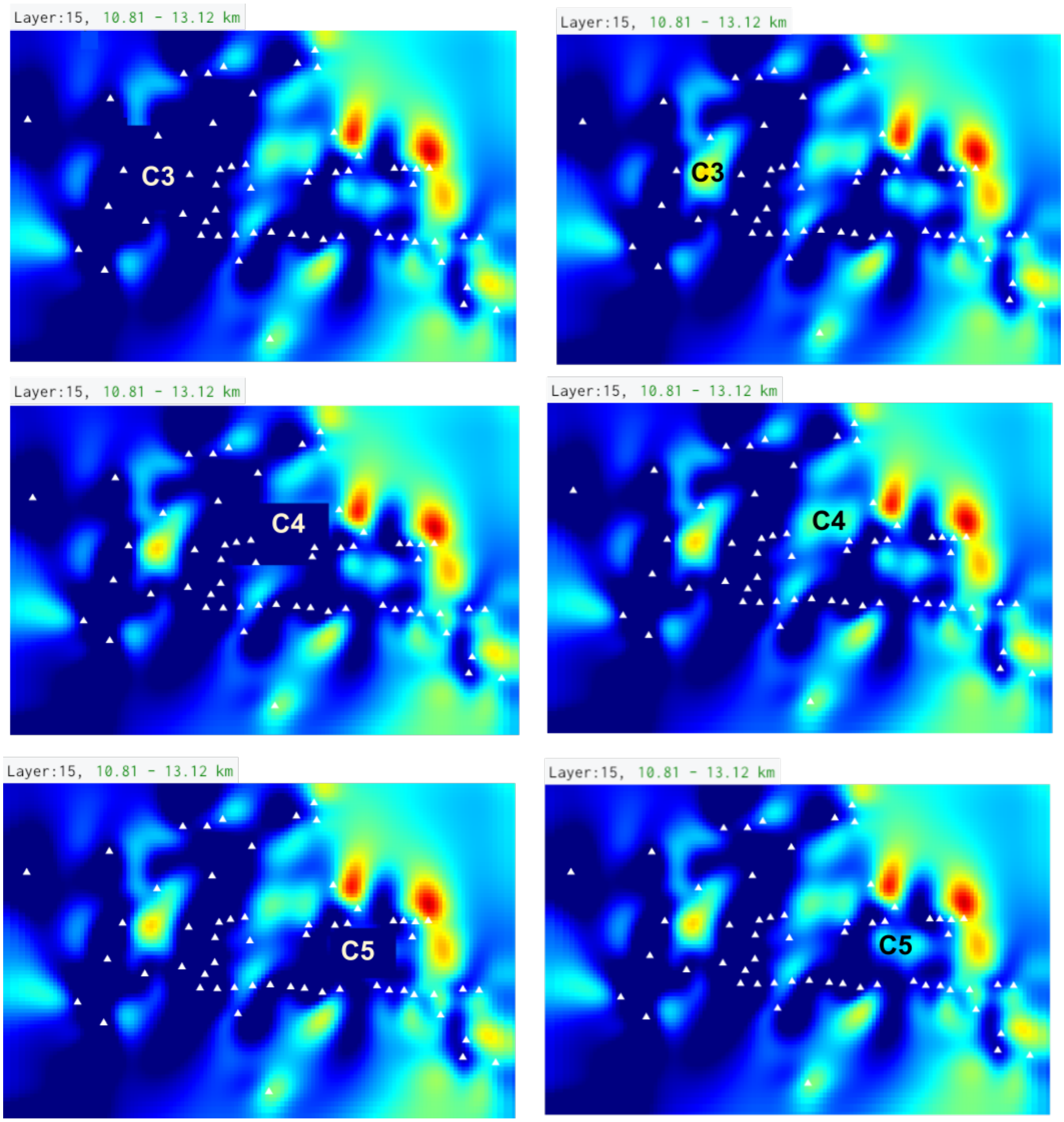

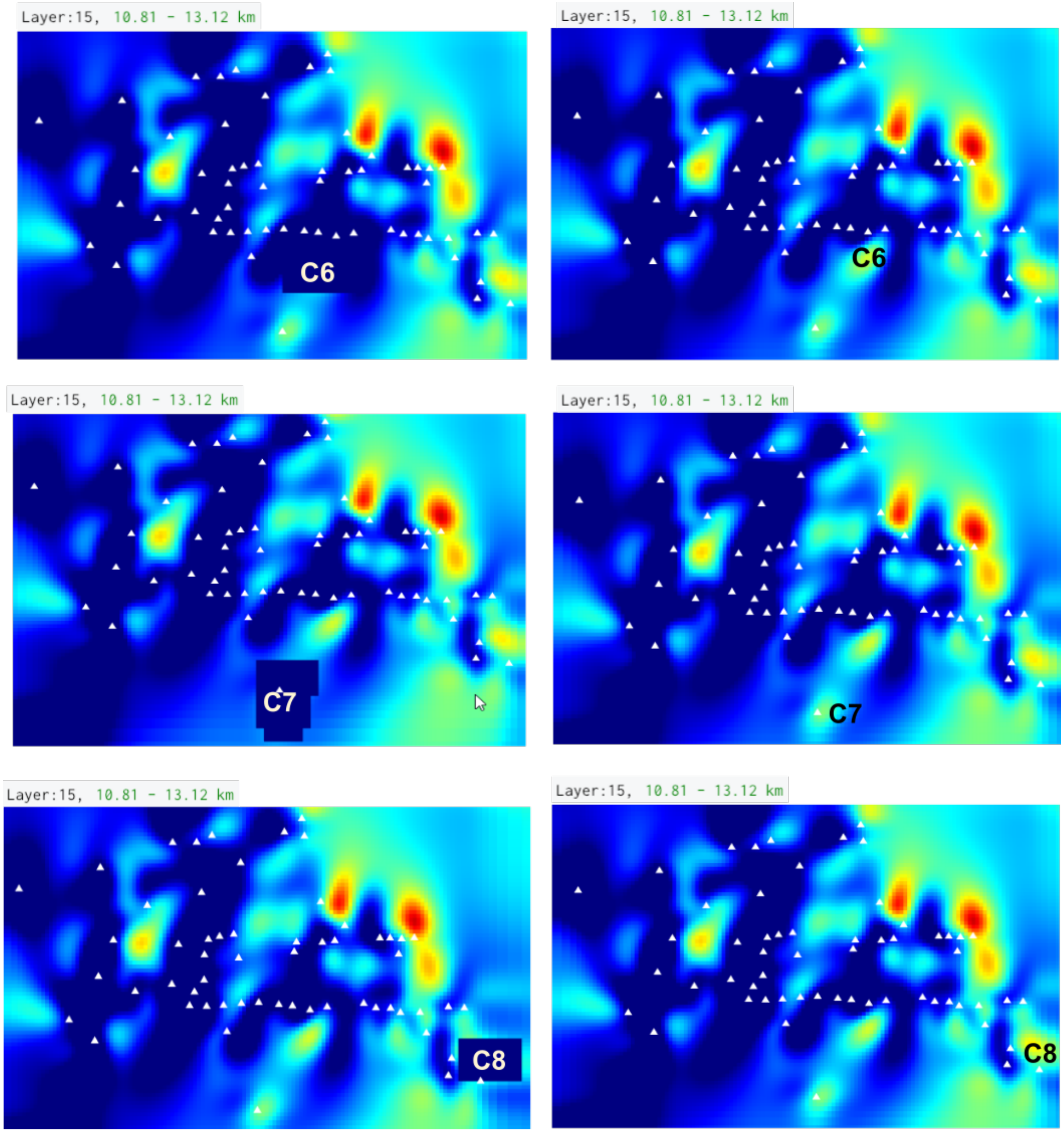

Figure S3.1. Horizontal layer at 10.81 – 13.12 km depth for the forward modeling tests (left) and model (right). The letters C3-C8 show the body of 1000 Ωm that replaced studied anomalies in the sensitivity tests[1]. We use the GMT software version 5.0 (https://www.generic-mapping-tools.org) for generating the map.

Station: pb15

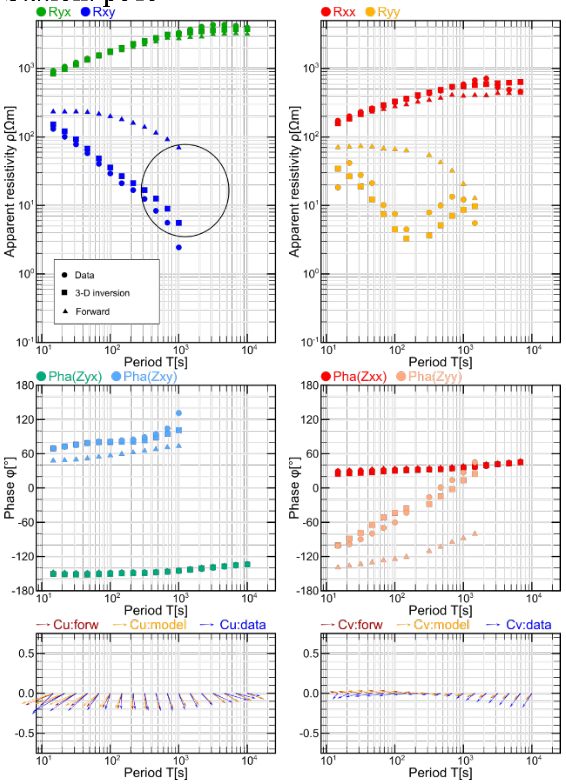

Station: s04

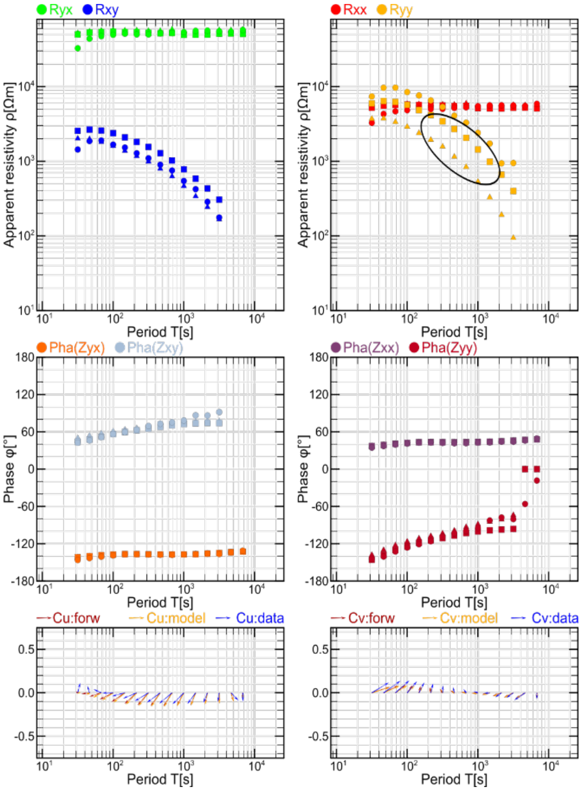

Station: s05

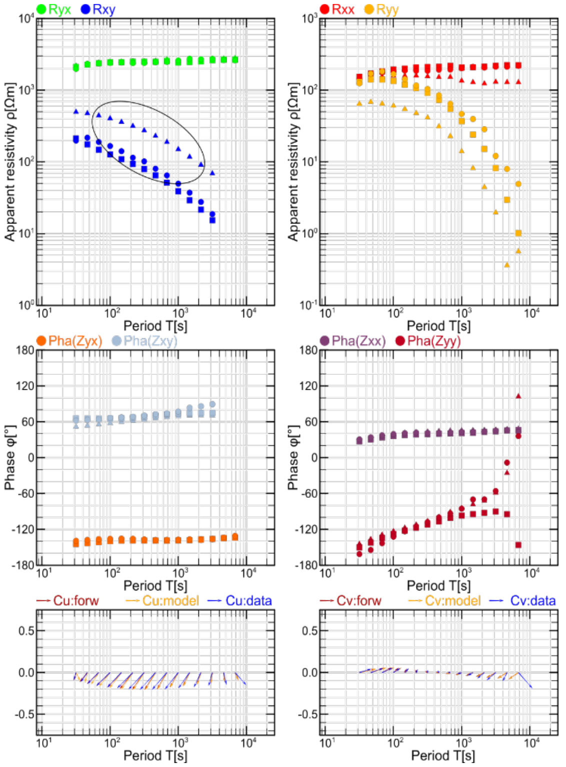

Station: s07

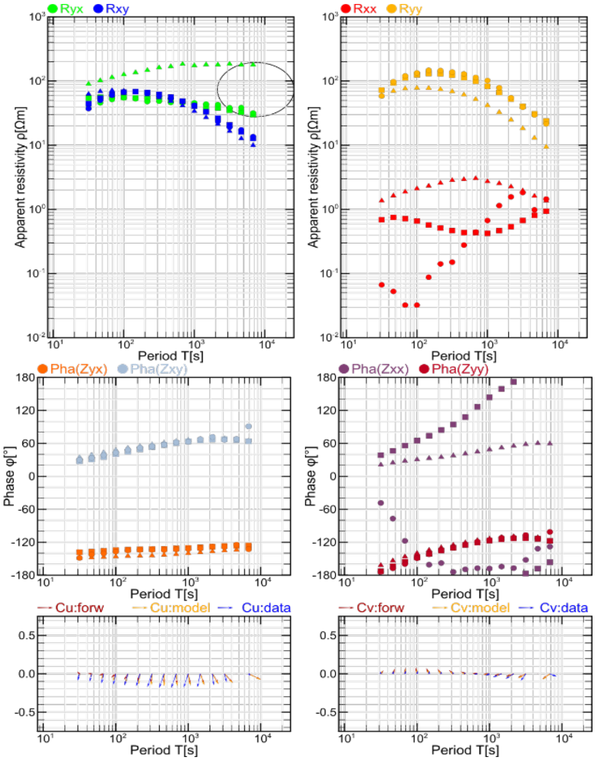

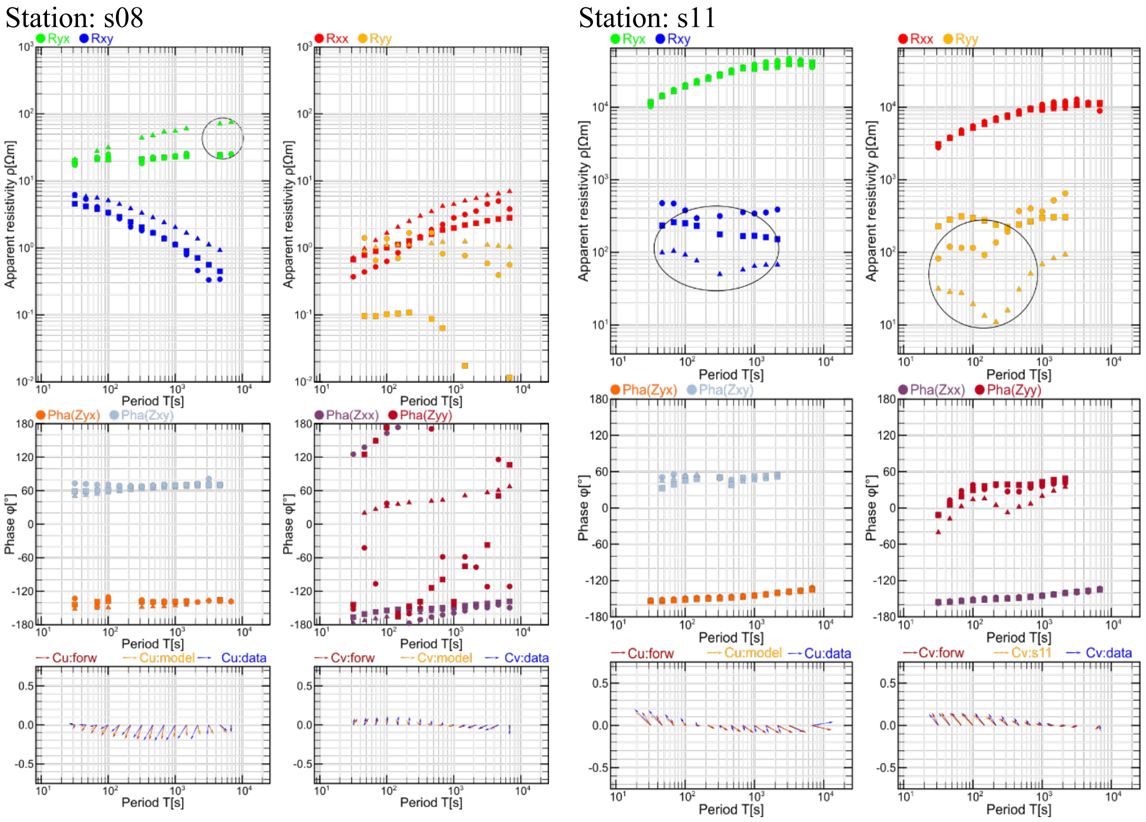

Figure S3.2. Apparent resistivity, phase and induction vectors Cu and Cv as functions of period. Comparison between the original data of 6 stations closest to C3 structure, result obtained from 3-D inversion and forward modeling done after removing of the structure. Black circles indicate TF (Transfer Function) components where maximum differences are observed. Induction vectors: red color represents the response after forward modeling tests; yellow color represents the response after inversion, blue represents measured data. We use the GMT software version 5.0 (https://www.generic-mapping-tools.org) for generating the map.

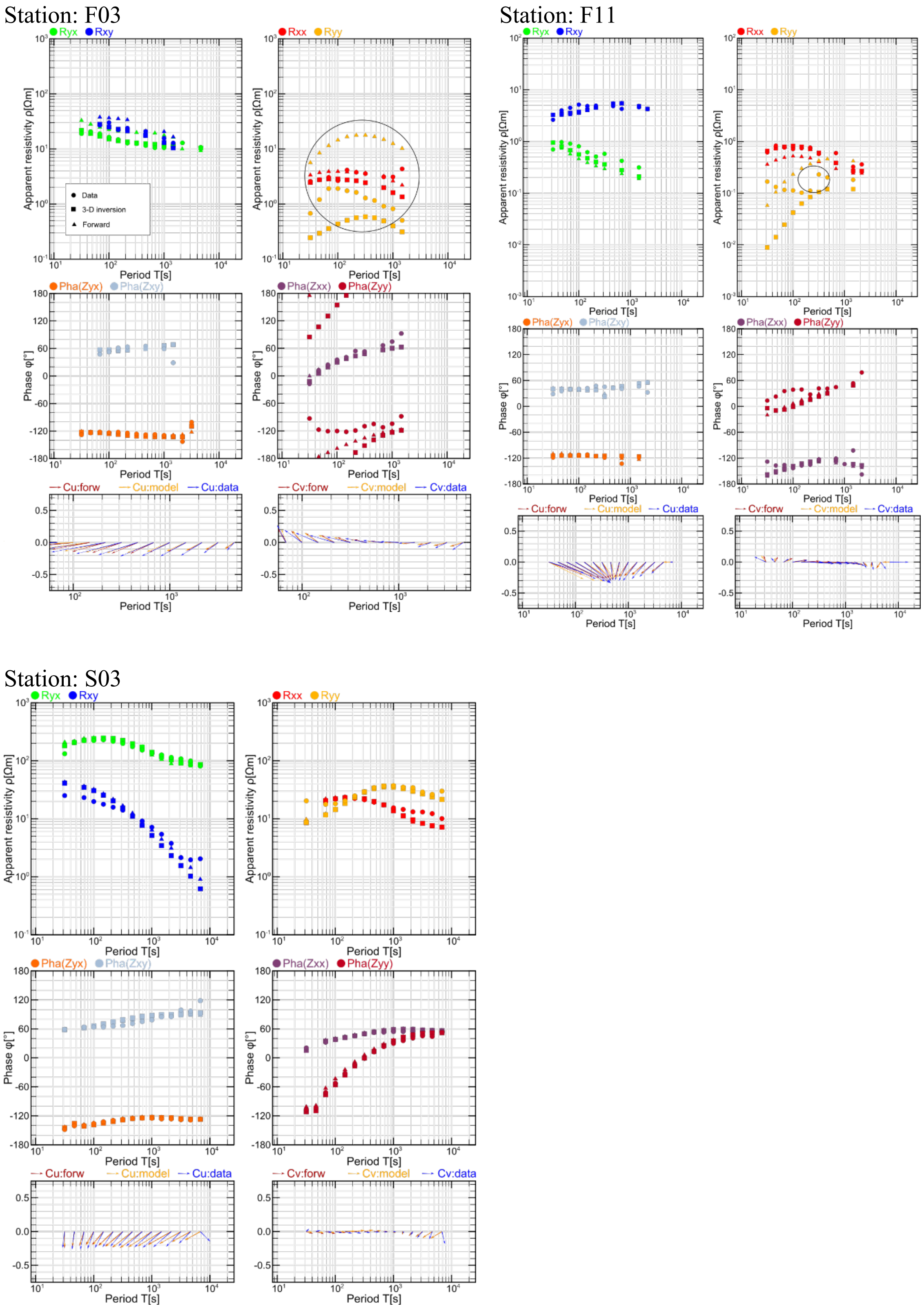

Figure S3.3. Apparent resistivity, phase and induction vectors Cu and Cv as functions of period. Comparison between the original data of 3 stations closest to C4 structure, result obtained from 3-D inversion and forward modeling done after removing of the structure. Black circles indicate s where maximum differences are observed. . Induction vectors: red color represents the response after

forward modeling tests; yellow color represents the response after inversion, blue represents measured data. We use the GMT software version 5.0 (https://www.generic-mapping-tools.org) for generating the map.

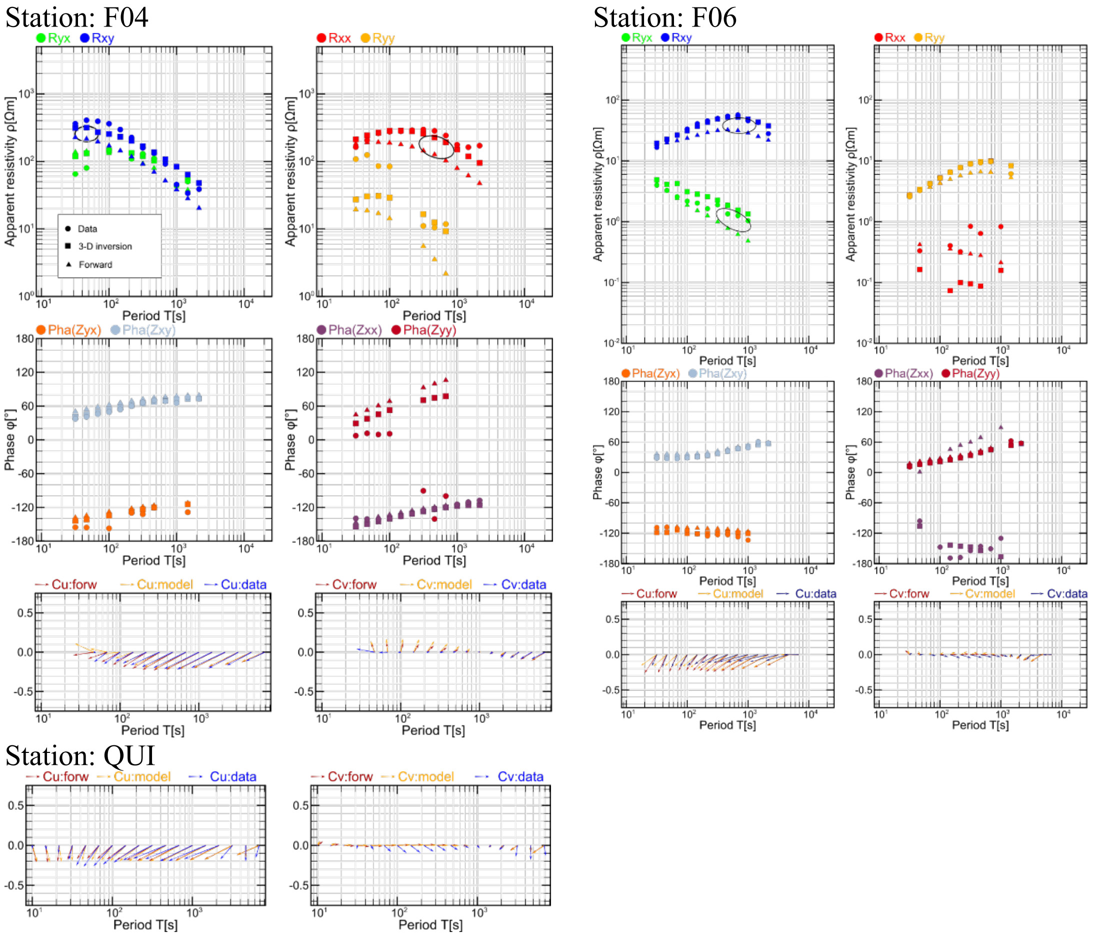

Figure S3.4. Apparent resistivity, phase and induction vectors Cu and Cv as functions of period. Comparison between the original data of 3 stations closest to C5 structure, result obtained from 3-D inversion and forward modeling done after removing of the structure. Black circles indicate TF components where maximum differences are observed. Induction vectors: red color represents the response after forward modeling tests; yellow color represents the response after inversion, blue represents measured data. We use the GMT software version 5.0 (https://www.generic-mapping-tools.org) for generating the map.

Station: SOP

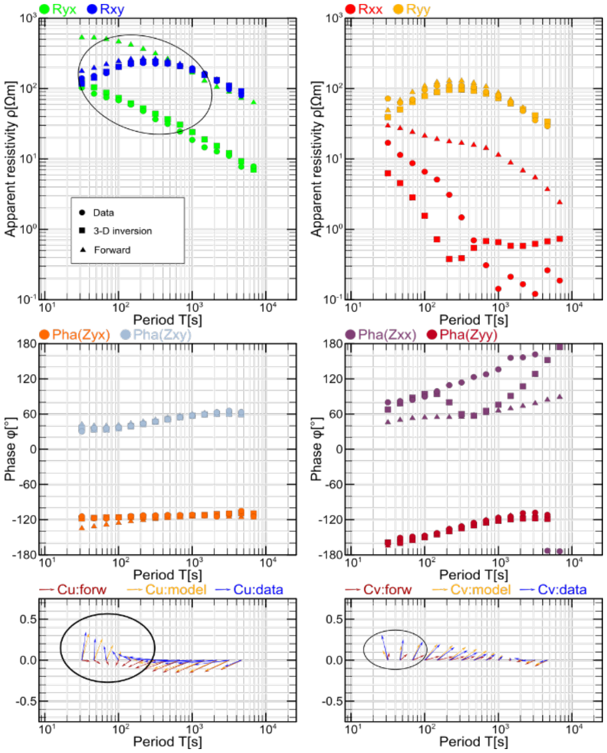

Station: PEI

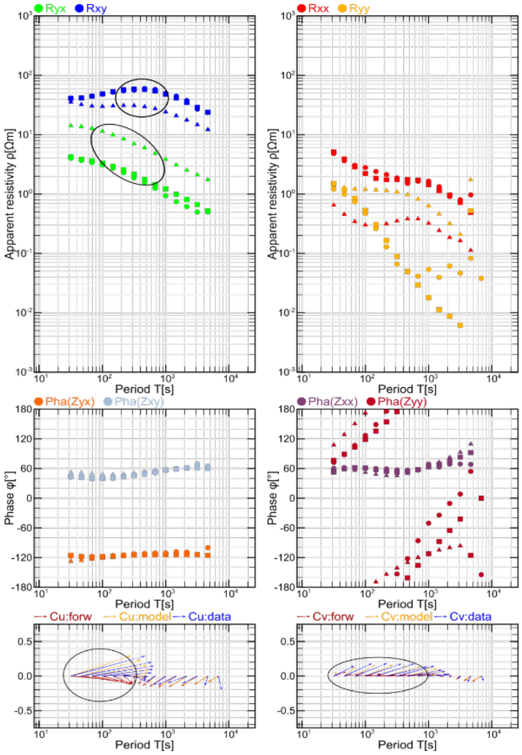

Station: PEU

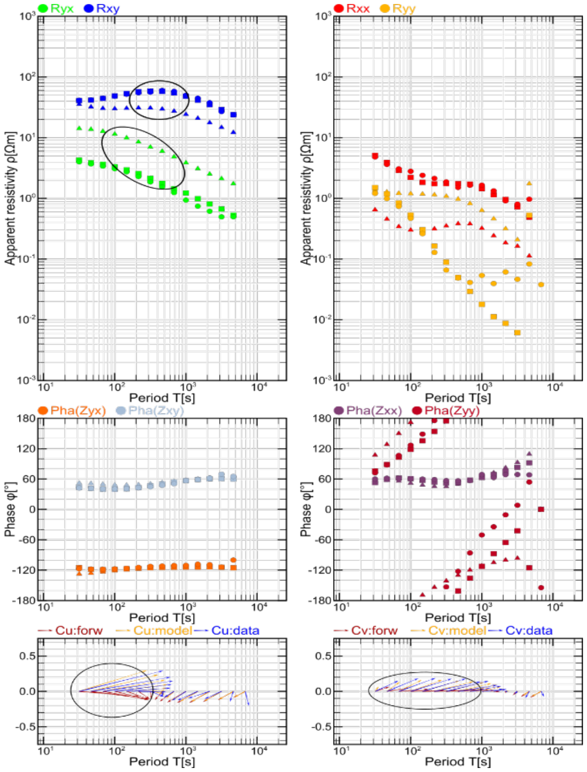

Station: SOC

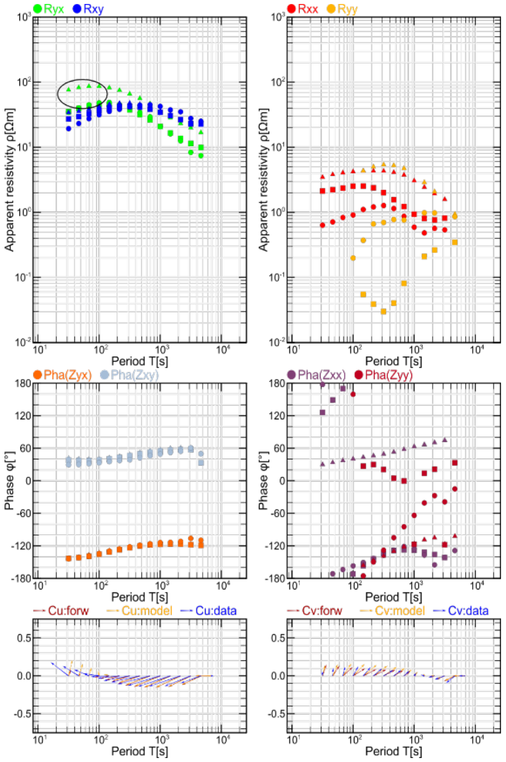

Figure S3.5. Apparent resistivity, phase and induction vectors Cu and Cv as functions of period. Comparison between the original data of 4 stations closest to C6 structure, result obtained from 3-D inversion and forward modeling done after removing of the structure. Black circles indicate TF components where maximum differences are observed. Induction vectors: red color represents the response after forward modeling tests; yellow color represents the response after inversion, blue represents measured data. We use the GMT software version 5.0 (https://www.generic-mapping-tools.org) for generating the map.

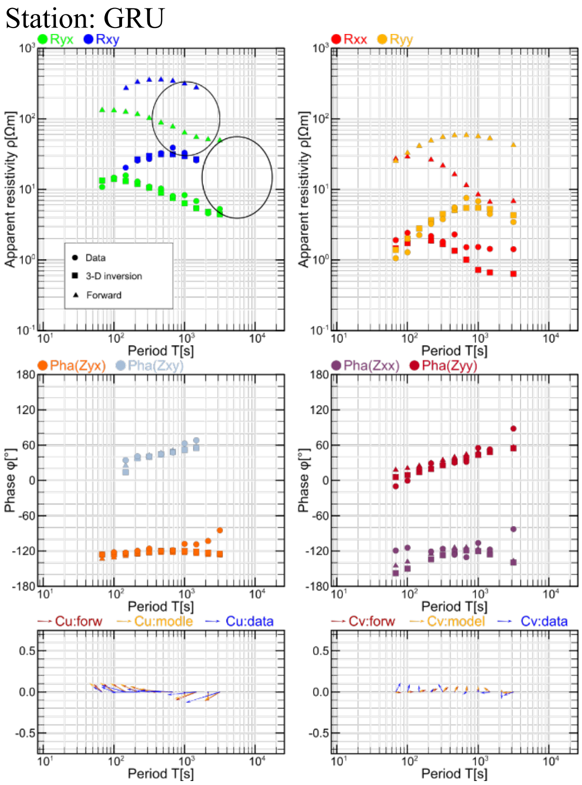

Figure S3.6. Apparent resistivity, phase and induction vectors Cu and Cv as functions of period. Comparison between the original data of the station closest to C7 structure, result obtained from 3-D inversion and forward modeling done after removing of the structure. Black circles indicate TF components where maximum differences are observed. Induction vectors: red color represents the response after forward modeling tests; yellow color represents the response after inversion, blue represents measured data. We use the GMT software version 5.0 (https://www.generic-mapping-tools.org) for generating the map.

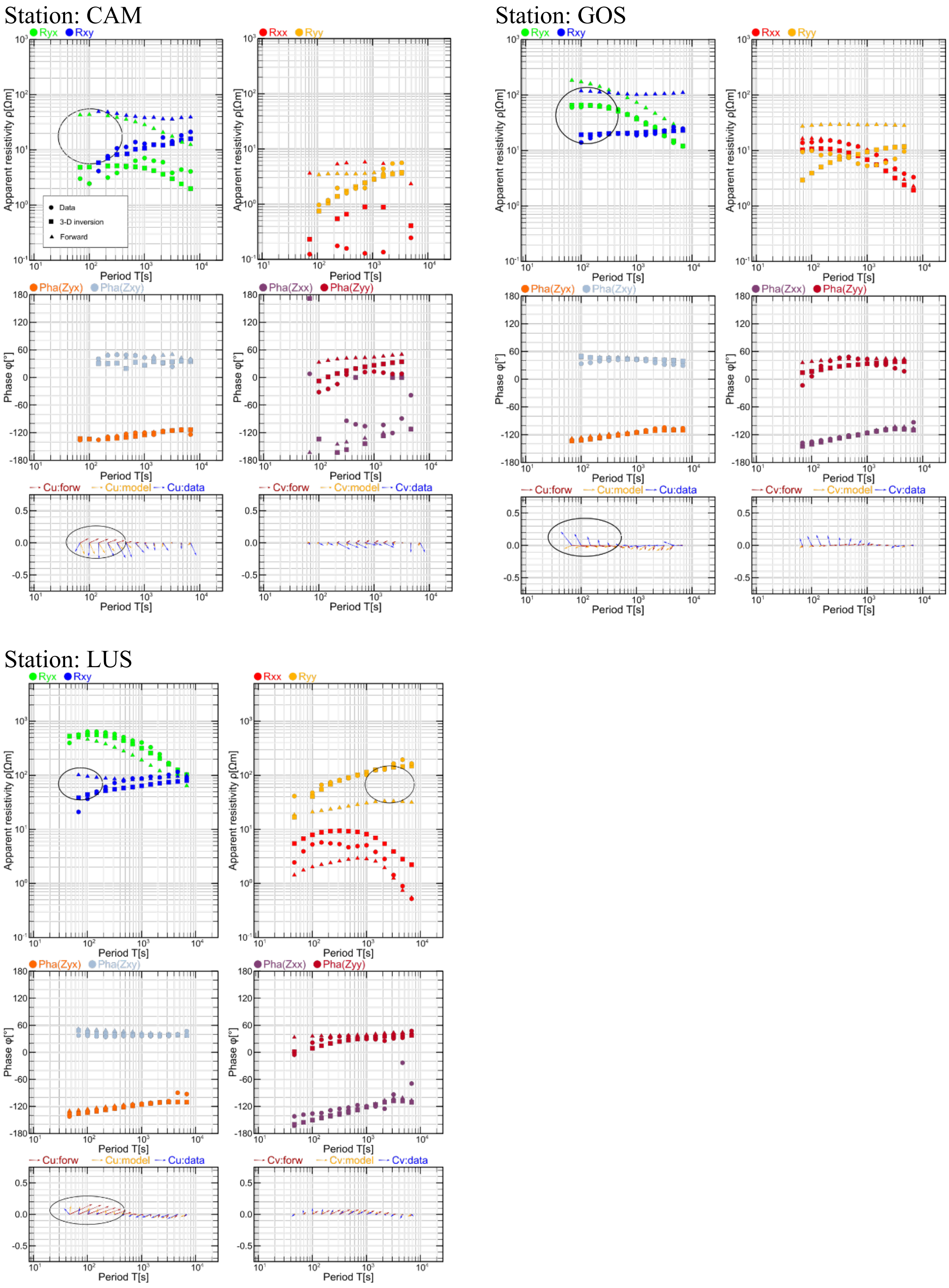

Figure S3.7. Apparent resistivity, phase and induction vectors Cu and Cv as functions of period. Comparison between the original data of 3 stations closest to C8 structure, result obtained from 3-D inversion and forward modeling done after removing of the structure. Black circles indicate TF components where maximum differences are observed. Induction vectors: red color represents the

response after forward modeling tests; yellow color represents the response after inversion, blue represents measured data. We use the GMT software version 5.0 (https://www.generic-mapping-tools.org) for generating the map.

1.2. Additional sensitivity tests

1.2.1 Sensitivity tests - the C2 extension outside the APVC

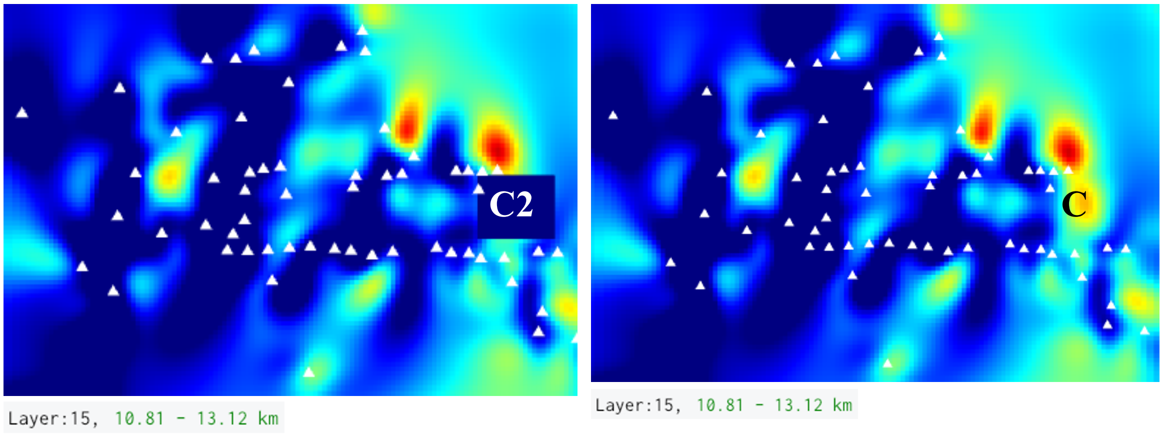

Figure S4.1. Horizontal layer at 10.81 – 13.12 km depth for the forward modeling tests (left) and model (right). The letter C2 shows the body of 1000 Ωm that replaced studied anomalies in the sensitivity tests. We use the GMT software version 5.0 (https://www.generic-mapping-tools.org) for generating the map.

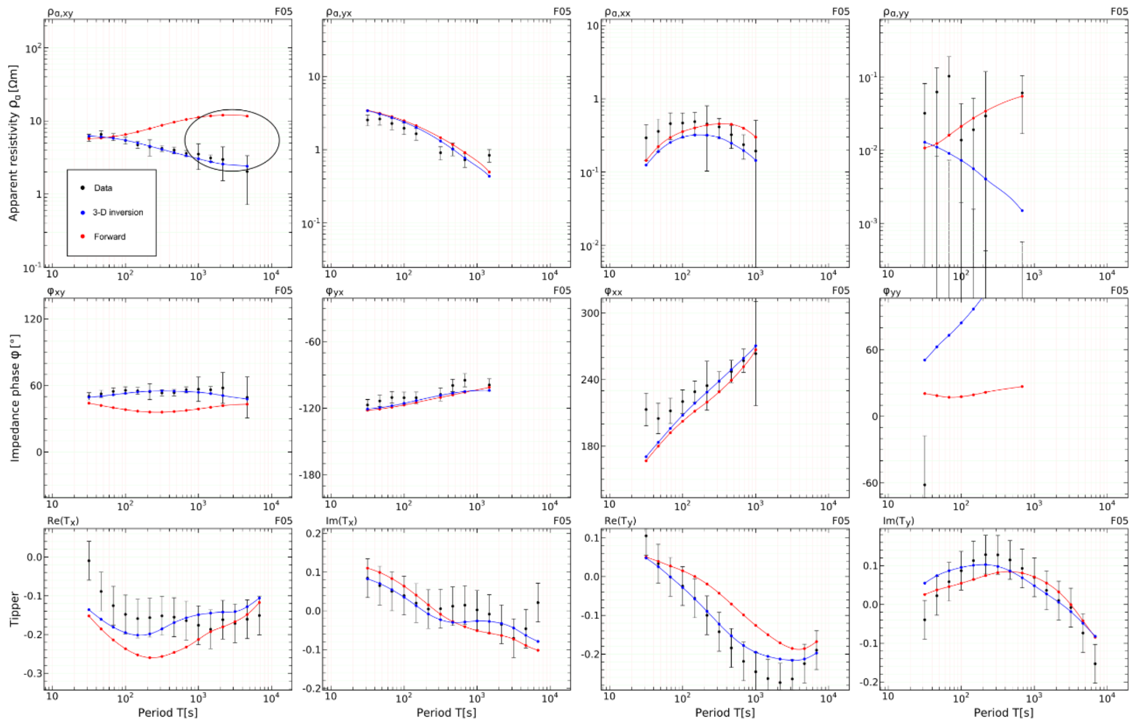

b)

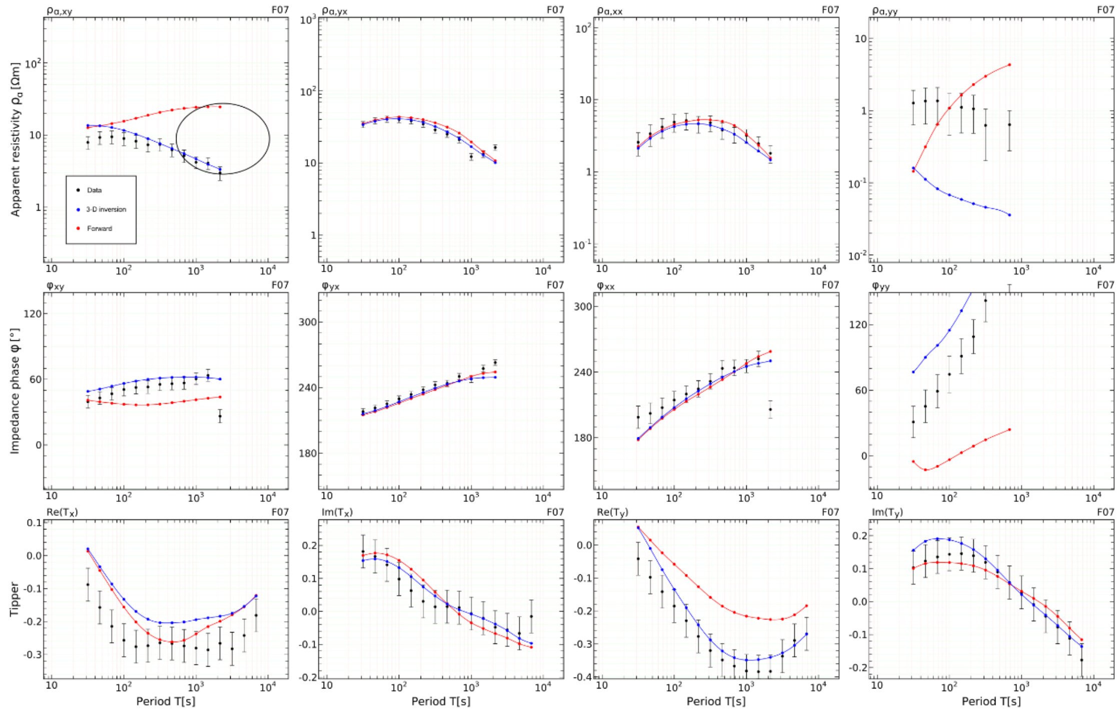

Figure S4.2. Apparent resistivity, phase and tippers as functions of period. Comparison between the original data of 2 stations closest to C2 structure, result obtained from 3-D inversion and forward modeling done after removing of the structure. Black circles indicate TF components where maximum differences are observed. Black points represent original data; blue points represent measured data; red points represent forward modeling done after removing of the structure C2. We use the GMT software version 5.0 (https://www.generic-mapping-tools.org) for generating the map.

1.2.2 Sensitivity tests - maximum depth of C2 structure.

a)

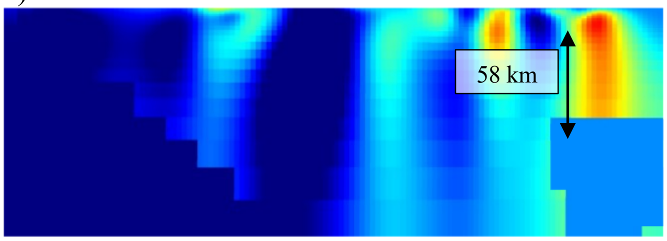

b)

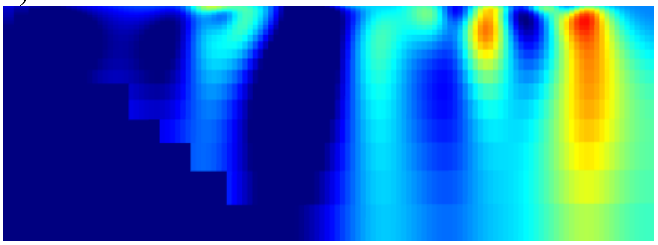

Figure S4.3. Cross-sections of 3-D electrical resistivity model at 23° S for the forward modeling tests (a) and model (b). The light blue area outlines the body of 100 Ωm that replaced the bottom of the studied anomaly from 58 km in the sensitivity tests. We use the GMT software version 5.0 (https://www.generic-mapping-tools.org) for generating the map.

a)

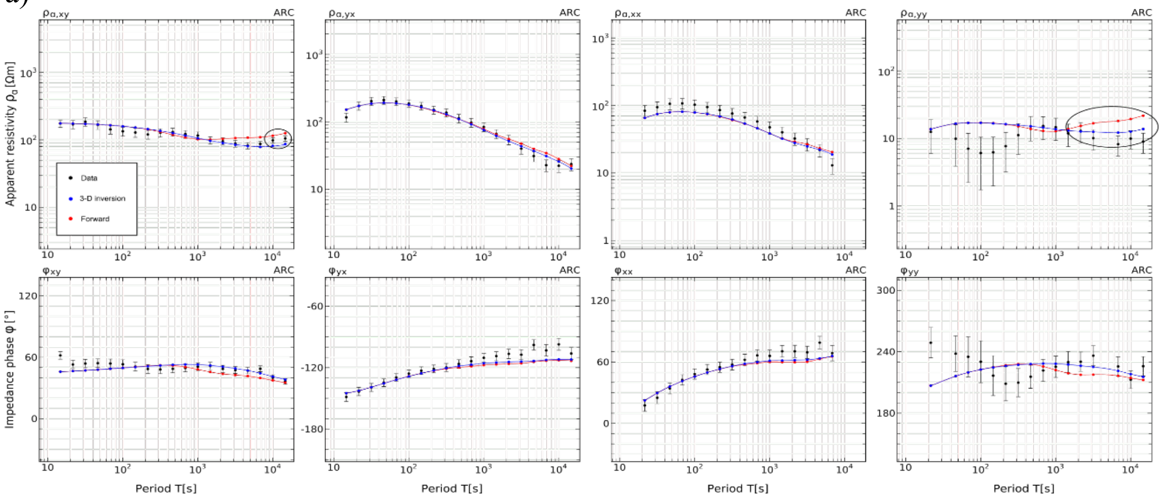

b)

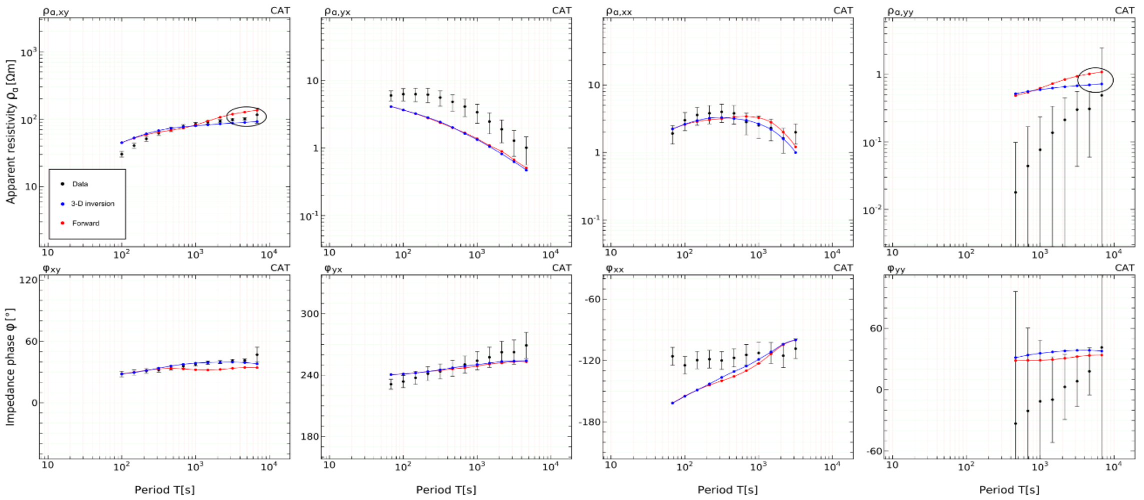

c)

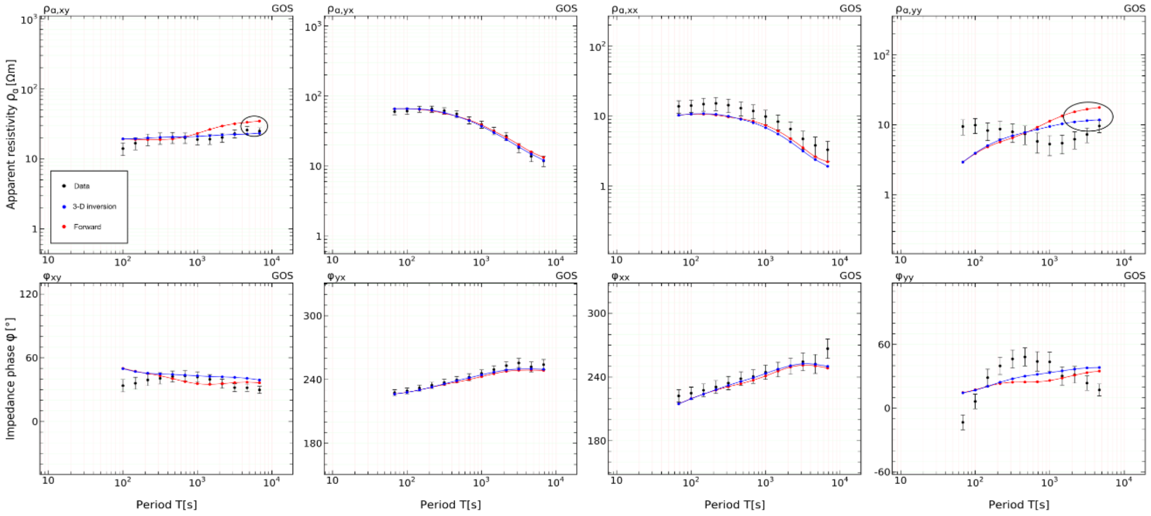

Figure S4.4. Apparent resistivity and phase as functions of period. Comparison between the original data of ARC (a), CAT (b) and s05 (c) stations, result obtained from 3-D inversion and forward modeling done after changing of the C2 structure. Black point – model data, red points – the bottom of the structure begins at 58 km and blue points - result obtained from 3-D inversion. We use the GMT software version 5.0 (https://www.generic-mapping-tools.org) for generating the map.

1.3 Sensitivity tests - maximum depth of structures C3.

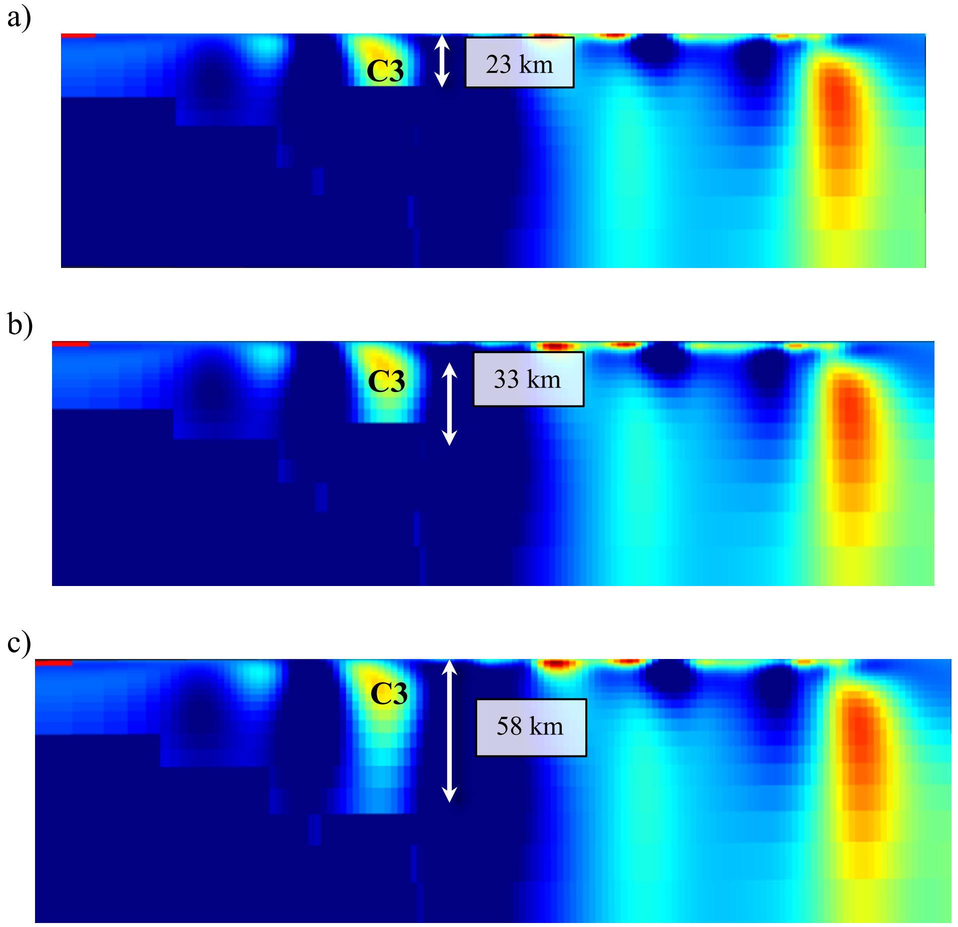

Figure S4.5. Cross-sections of 3-D electrical resistivity model at 23° S. The body of 1000 Ωm replaces the bottom of the studied anomaly for 23 km (a), 33 km (b) and 58 km (c). We use the GMT software version 5.0 (https://www.generic-mapping-tools.org) for generating the map.

a)

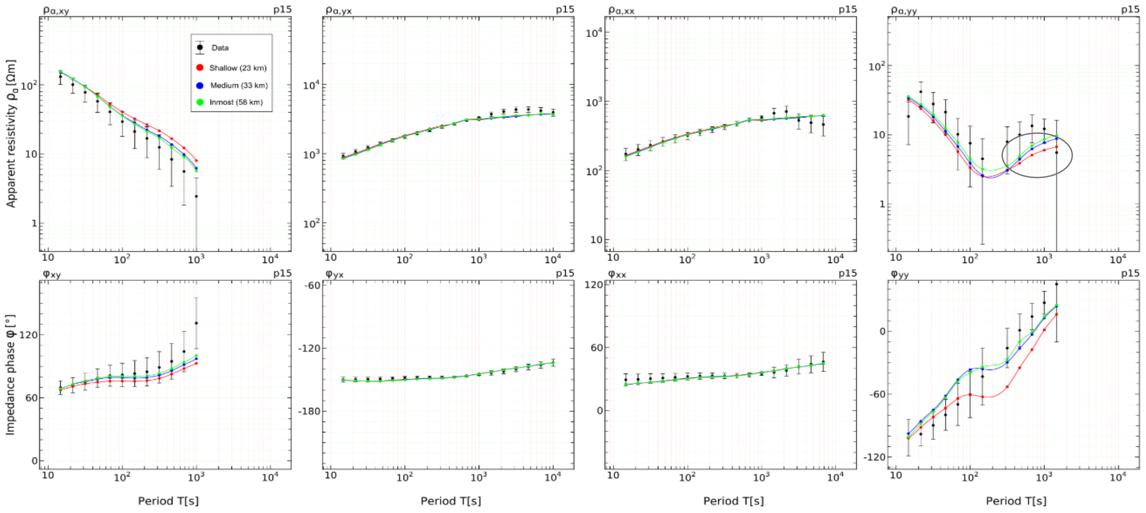

ρa,xy
p15
ρa,yx
p15
ρa,xx
p15
ρa,yy
p15
Data
Shallow (23 km)
Medium (33 km)
Inmost (58 km)
Apparent resistivity ρa [Ωm]
φxy
p15
φyx
p15
φxx
p15
φyy
p15
Impedance phase φ [°]
Period T[s]
Period T[s]
Period T[s]
Period T[s]

b)

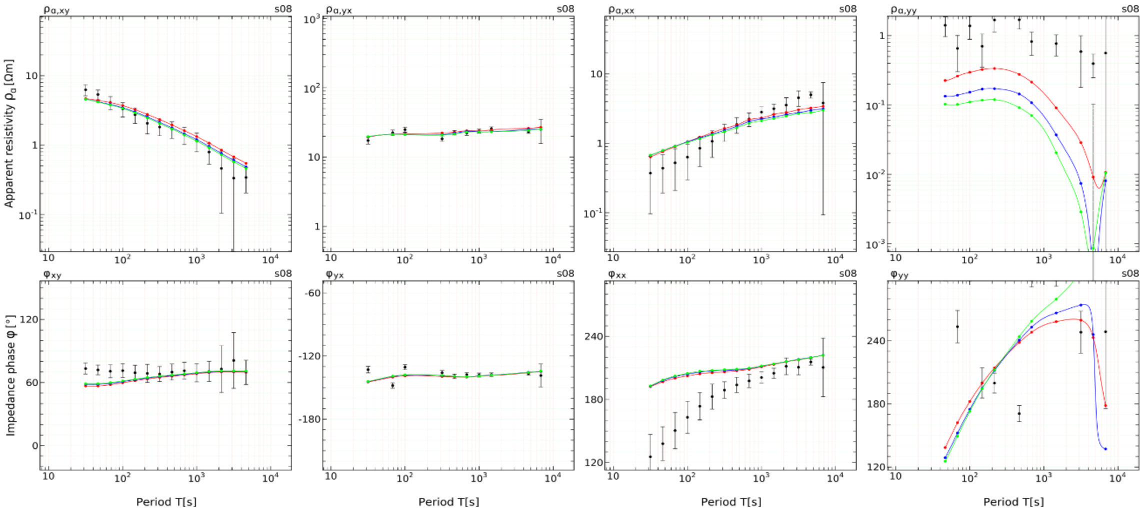

ρa,xy
s08
ρa,yx
s08
ρa,xx
s08
ρa,yy
s08
Apparent resistivity ρa [Ωm]
φxy
s08
φyx
s08
φxx
s08
φyy
s08
Impedance phase φ [°]
Period T[s]
Period T[s]
Period T[s]
Period T[s]

c)

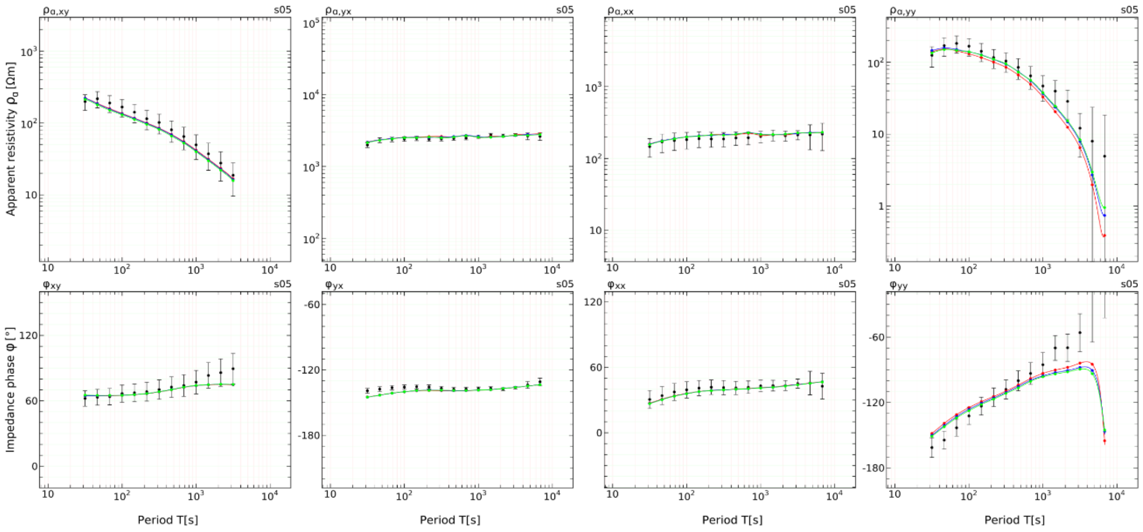

Figure S4.6. Apparent resistivity and phase as functions of period. Comparison between the original data of p15 (a), s08 (b) and s05 (c) stations, result obtained from 3-D inversion and forward modeling done after changing of the C3 structure. Black point – model data, red points – the bottom of the structure begins at 23 km, blue points - the bottom of the structure begins at 33 km, green points - the bottom of the structure begins at 58 km. We use the GMT software version 5.0 (https://www.generic-mapping-tools.org) for generating the map.

1.4 Sensitivity tests - maximum depth of structures C4.

a)

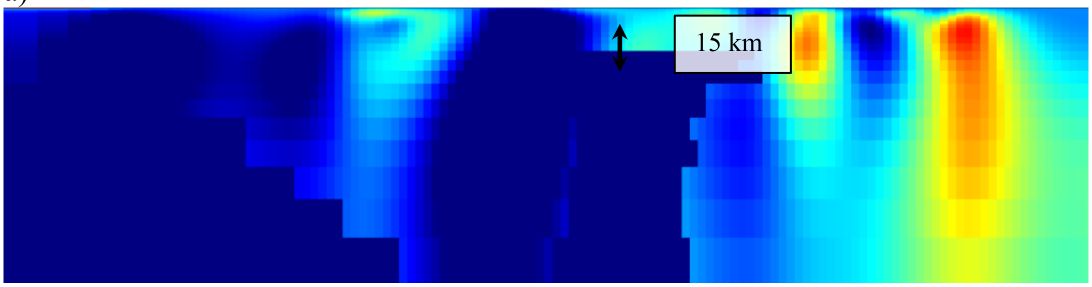

b)

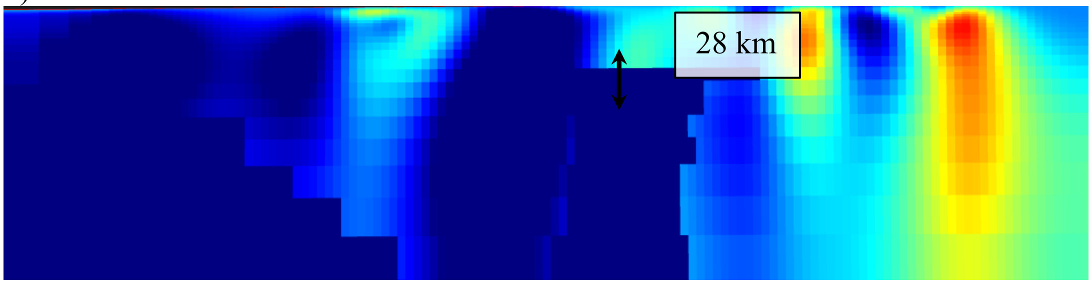

c)

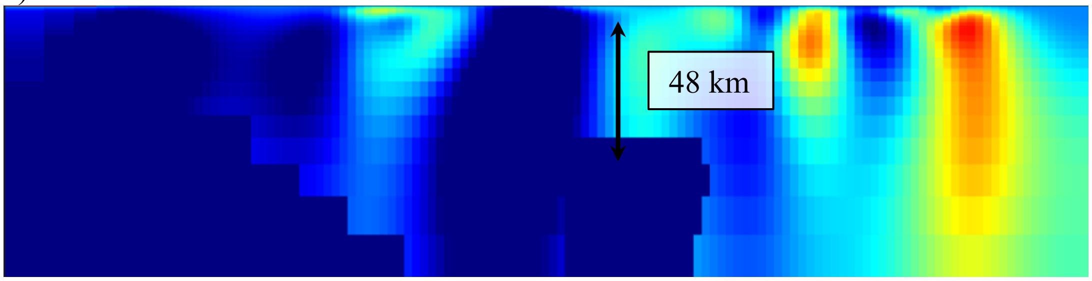

Figure S4.7. Cross-sections of 3-D electrical resistivity model at 22.85° S. The body of 1000 Ωm replaces the bottom of the studied anomaly for 15 km (a), 28 km (b) and 48 km (c). We use the GMT software version 5.0 (https://www.generic-mapping-tools.org) for generating the map.

a)

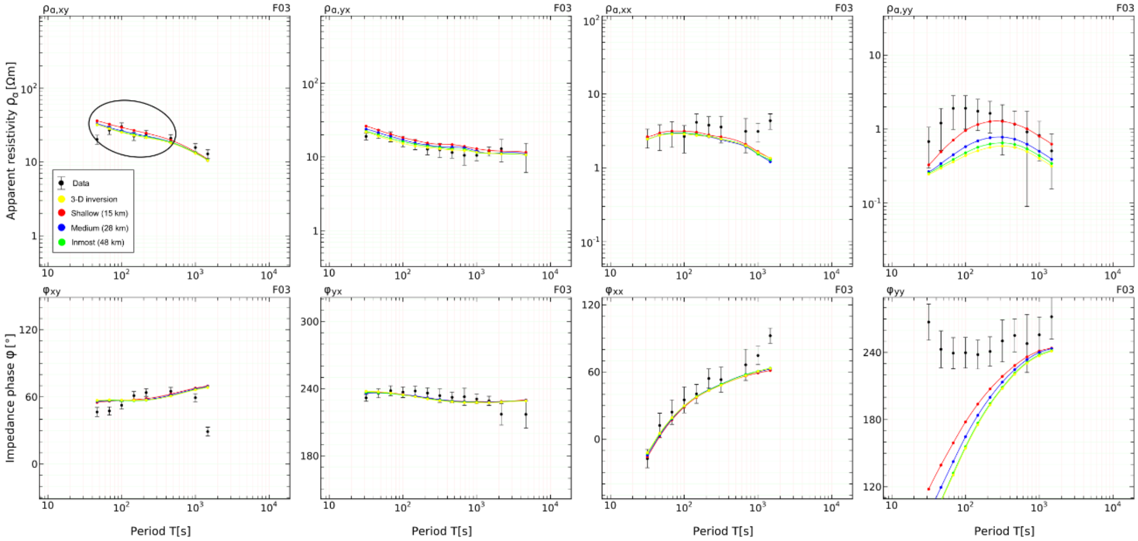

b)

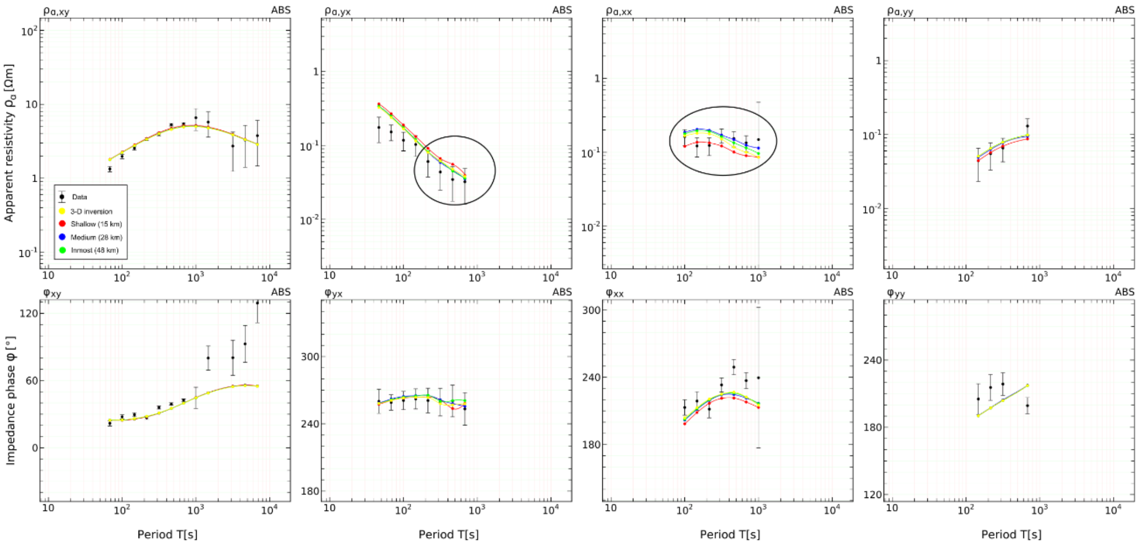

Figure S4. 8. Apparent resistivity and phase as functions of period. Comparison between the original data of F03 (a) and ABS (b) stations, result obtained from 3-D inversion and forward modeling done after changing of the C4 structure. Black point – model data, red points – the bottom of the structure begins at 15 km, blue points - the bottom of the structure begins at 28 km, green points - the bottom of the structure begins at 48 km. We use the GMT software version 5.0 (https://www.generic-mapping-tools.org) for generating the map.

2. Seismological tomography

2.1 starting model: Comte et al. (2016)[6] incorporated surface wave observations to adjust the model before the joint inversion. For the starting model for the surface wave only (SWO) inversion, they began with a body wave only (BWO) inversion that started with an adaptation of the 1-D model of Husen et al. (1999)[2] for the Antofagasta region (Fig. 8). The resulting 3-D BWO model for Vp and Vp/Vs, obtained after 13 iterations, was averaged laterally to obtain a 1-D estimate of Vs. This step ensures some compatibility with the body waves and provides a means for determining appropriate values for regularization and thresholds for data misfit. Because surface wave inversions tend to retain biases introduced by interfaces like the Moho, they smoothed the transition from crustal to mantle velocities over a range of depths (40–70 km) suggested by previous estimates of crustal thickness in this area[3-5]. This 1-D Vs model[6] was then used as a starting model for the SWO inversion.

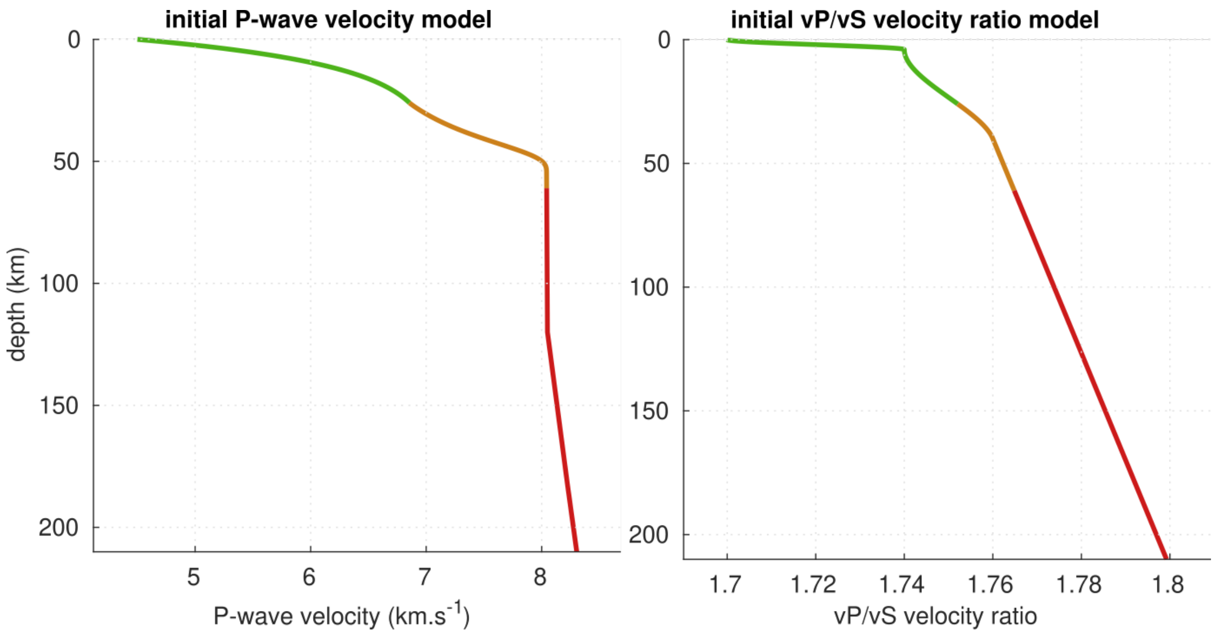

Figure S5. Initial velocity model. It is a 1D continuous model, function of depth, for both vP and vP/vS parameters. vP crust model (green line) is a simple function starting at 4.5 km/s at sea-level and rapidly increasing toward an asymptotic value of 7.1 km/s, corresponding to the lower crust. Mantle velocity (red line) corresponds to the AK135 velocity model[7]. The Moho velocity jump is modeled by a smooth transition (orange line) between 30 and 60 km, corresponding roughly to the Moho depth variation in the region. vP/vS values for the crust are deduced from Poisson's measurement by Ji *et al.* (2018)[8] and from the AK135 in the mantle. We use the GMT software version 5.0 (https://www.generic-mapping-tools.org) for generating the map.

2.2 Model parametrization: The subsurface was parameterized by Comte et al. (2016)[6] specifying P and S wave speeds on a 3-D grid of nodes spaced at 5 km intervals in depth and latitude and about the same distance in longitude (increments in longitude are everywhere 0.04717°). Intragrid wave speeds are determined by trilinear interpolation. Body wave traveltimes within the medium are calculated using a 3-D Eikonal equation solver in a spherical (Earth-centred) coordinate system. Surface wave phase delay times at a given frequency ω in each 3-D Vs model are determined by first assuming that such a model can be constructed by combining 1-D models at each areal grid point[9]. With this assumption, they calculated phase velocities c and partial derivatives ∂c/∂Vs for the 1-D model at each areal point using the locked-mode method of Gomberg and Masters (1988)[10]. The phase delay times between any two points were then calculated by integrating the reciprocal of the phase velocity along the great circle path between them.

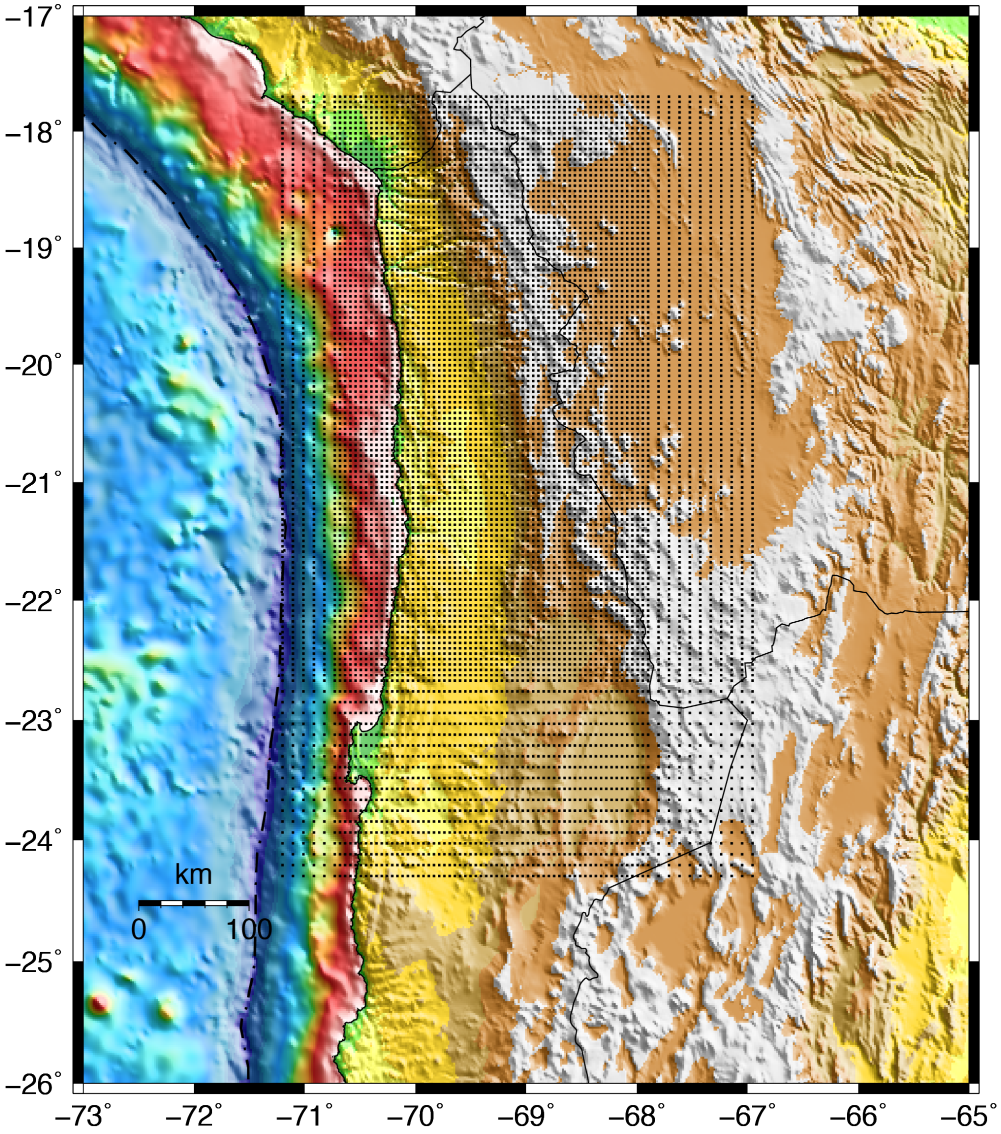

Figure S6. Geometry of the experiment. The black dots indicate the model-nodes of the 3D seismological tomography model. We use the GMT software version 5.0 (https://www.generic-mapping-tools.org) for generating the map.

2.3. Data processing info: Most of the ambient noise analyzed by Comte et al. (2016)[6] was recorded by 18 broad-band stations operated by the Integrated Plate Boundary Observatory Chile (IPOC), CSN and ONEMI throughout 3 yr (2012 January 1–2014 December 31). This primary dataset was supplemented by recordings from 29 broad-band stations deployed by the DGF, CSN and ONEMI near Pisagua for 4 months (2014 March 29–2014 July 31) and 12 broad-band stations of the Iquique network operated by GFZ Potsdam for 7 months (2012 January 10–2013 September 5). The 59 total stations in these combined networks (Fig. 3) provide 1422 contemporaneous station pairs from which Green's functions may be estimated. For the most part they followed the pre-processing steps described in Bensen et al. (2007)[11] to generate estimated Green's functions (EGFs) from the

vertical component of cross-correlated noise, using a version of the CU-Boulder ANCC software,1 modified to permit sample rates and frequency bands appropriate for our data. Running-mean normalization was used to reduce contamination by coherent signals (mostly from earthquakes), and we pre-processed a band between periods of 2 and 150 s. Cross-correlations for each contemporary station pair were generated for each day of record and then stacked over the duration of co-recording. In an attempt to improve the signal-to-noise ratio (SNR) of the final stack, they iteratively removed daily correlations that deviated by more than one standard deviation from the overall stack.

The initial body wave data set, used by Comte et al. (2016) consisted of P and S arrival times from 33 351 events recorded throughout 25 yr by a variety of networks comprising 360 stations (Fig S4). From this combined data set, Comte et al. (2016)[6] selected earthquakes that were recorded by at least 10 stations and with predicted arrival times within an initial outlier residual threshold of the larger of 2 s and 10 percent of the total traveltime. Application of these criteria resulted in a reference data set of 11874 events with 110640 P and 106680 S wave arrival times. These arrival times and their associated uncertainties are estimated manually. Nominal uncertainties for P wave arrival times are between 0.1 and 0.5 s while those for S waves typically are about twice that amount. Outliers in the surface wave data set were identified and removed during the creation of phase velocity maps as part of the noise bias analysis. For body waves, data quality criteria were enforced at each iteration to eliminate potential outliers and to disqualify less well-constrained hypocentres. Thresholds for outlier identification were applied at each iteration, and observations that exceed these thresholds were disqualified for that iteration. These thresholds are generous at the start but gradually reduced over several iterations to the larger of 0.5 seconds or 5 percent of the traveltime.

For the case of the Pasten-Araya et al., (2021)[12] model, the tomography model is determined following a modern approach based on Tarantola and Valette (1982)[13], described in Araujo *et al.* (2021)[14]. The dataset in a modified version of the CSN catalogue (Centro sismológico Nacional, Universidad de Chile) merged with local experiment data[6] and cleaned using several tools such as Wadati and localization anomalies analysis. The dataset is made of 26,000 earthquakes registered by 150 stations, 225,000 P- and 209,000 S-waves arrival-times. The model is a set of vP and vP/vS values defined at each node of a regularly spaced grid covering the study area, each node is separated by 3 km horizontally and 1.5 km vertically. The model spreads over 410*420 km^2 horizontally and from the surface to 250 km at depth, leading to 3.251.488 nodes.

Travel-times are determined by a 3D Eikonal solver[15] on a thinner grid made of cubic cells of dimention 0.75 km, interpolated from the inversion grid at each iteration. Inversion is performed by a least-square approach[16], controlled by smoothing and damping parameters, determined based on L-curve analysis.

2.4 model uncertainties: As no single type of trail inversion provides conclusive evidence about the quality of an image, a variety of tests with both real and synthetic data were performed by Comte et al. (2016)[6] to estimate the level of robustness of certain features in the preferred wave speed model. To account for the effects of observational uncertainty, each synthetic data set was modified by random noise using a normal distribution with standard deviations that reflect the expected uncertainties in the actual data.

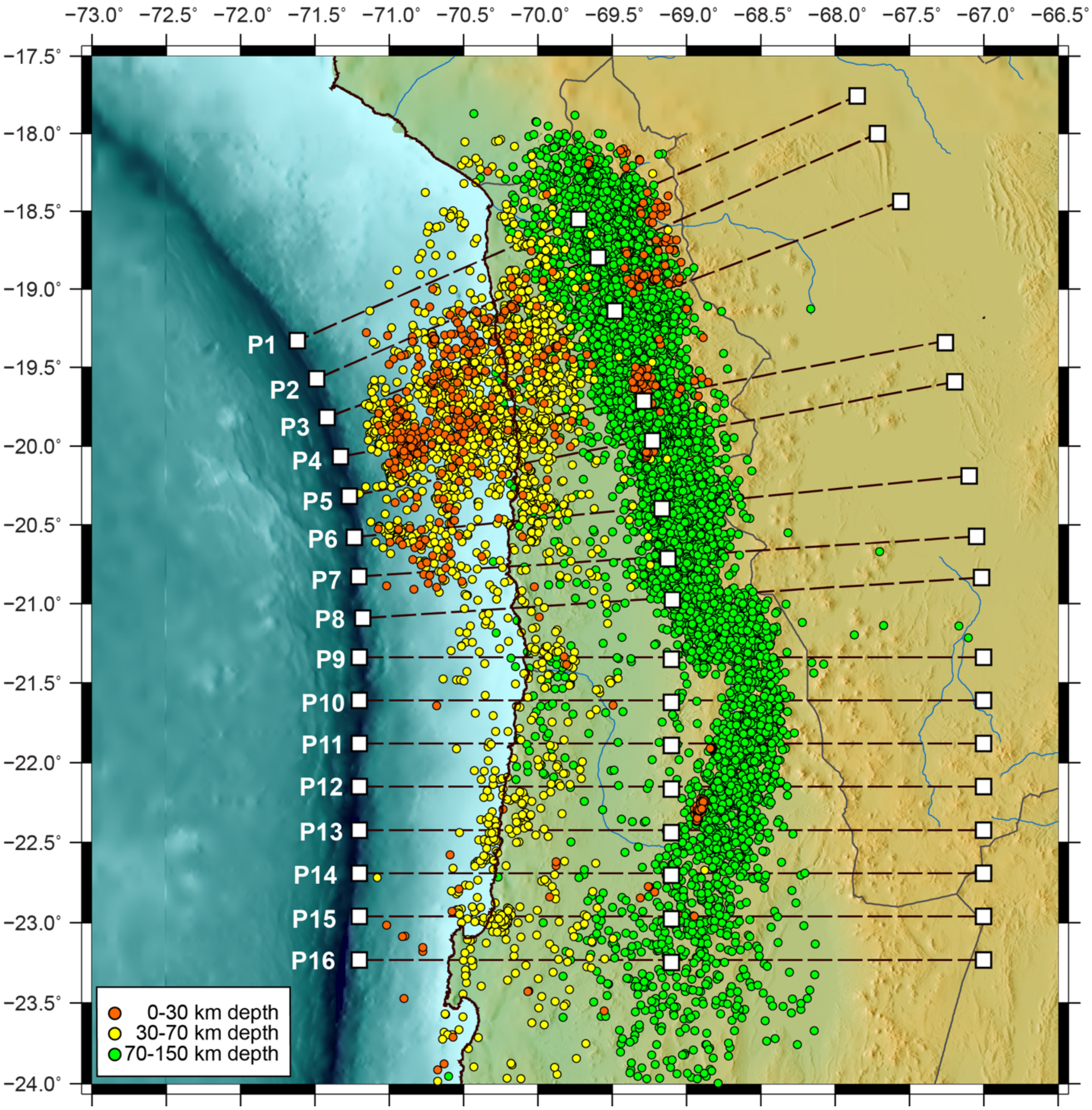

Figure S7. Locations of earthquakes used by Comte et al., (2016), indicated by circles filled with colors corresponding to depth ranges shown in the insert at the lower left. White squares joined by dashed lines and labeled P1-P16 locates the ends and midpoint origins (x=0) in the cross sections shown in Fig S9. We use the GMT software version 5.0 (https://www.generic-mapping-tools.org) for generating the map.

2.5 checkboard and resolution tests: A standard approach to estimating resolution in arrival time inversion is the 'checkerboard' test that attempts to recover alternating positive and negative perturbations in abutting prisms. Comte et al. (2016)[6] conducted versions of this test in which ±5 percent perturbations relative to a background 1-D model were applied to individual rectangular prisms that are 20 × 20 km in area and 10 km in depth. Results of these tests show that, in general, regions sampled with at least 10 ray paths are reasonably well recovered (Fig. S5). Moreover, despite recovered perturbations being smoothed at every iteration, the amount of smearing outside the anomaly is small over most of the region. In the test shown here (Fig. S6), Vp and Vs are both perturbed by ±5 percent, with a zero Vp/Vs anomaly. The near-identical recovery of Vp and Vs in this example shows that Vs can be resolved when solving for Vp/Vs rather than Vs directly, even when Vp/Vs itself is associated with a null signal.

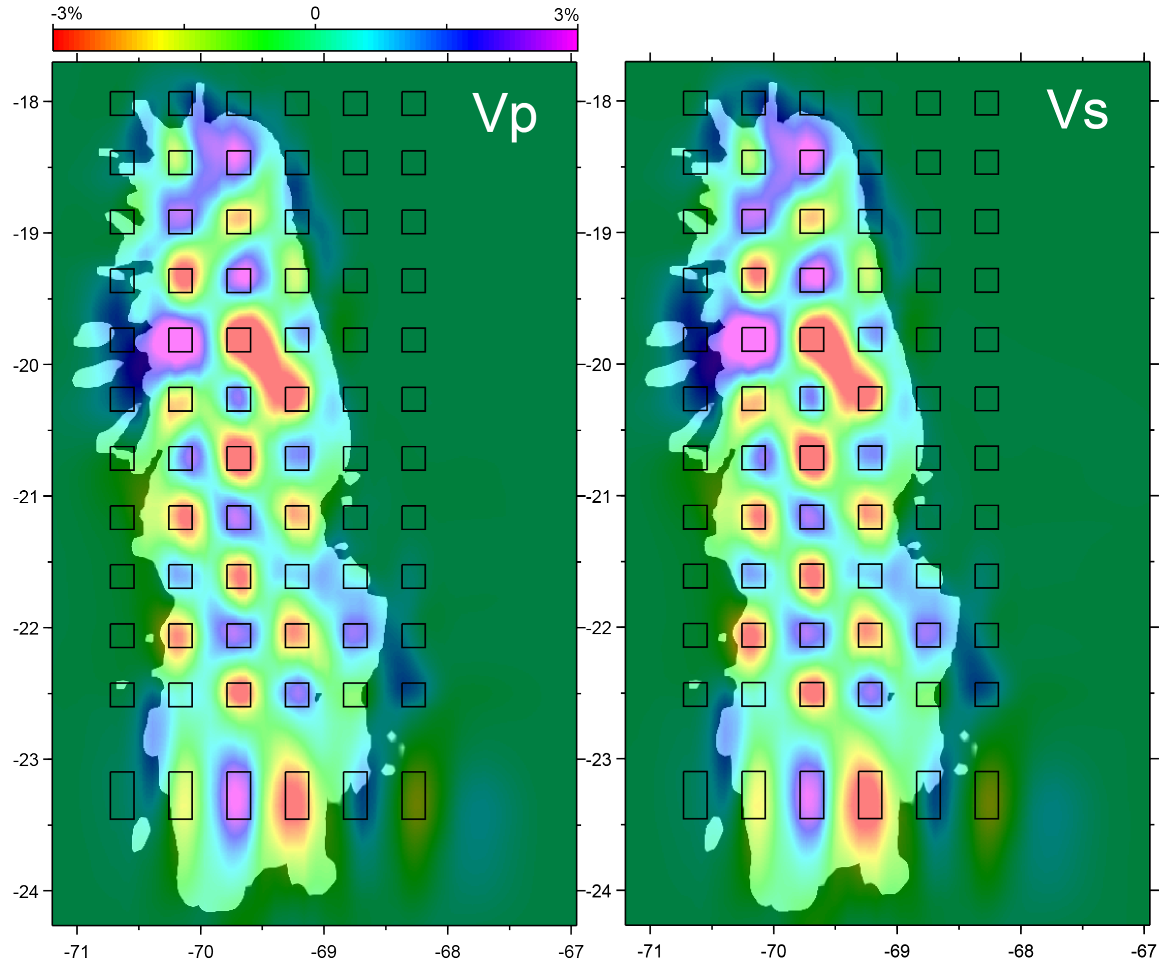

Figure S8. Checkerboard reconstruction test at 50 km depth for V_p (left) and Vs (right). Values are indicated by the color palette in the upper left part of the figure. The plots are shaded to reflect density of sampling, with the brightest regions corresponding to regions samples by at least 10 ray

paths. The rectangles locate the bounds of the prisms that are perturbed from the background 1D model. Note that the reconstruted images are nearly identical for V_p and V_s. We use the GMT software version 5.0 (https://www.generic-mapping-tools.org) for generating the map.

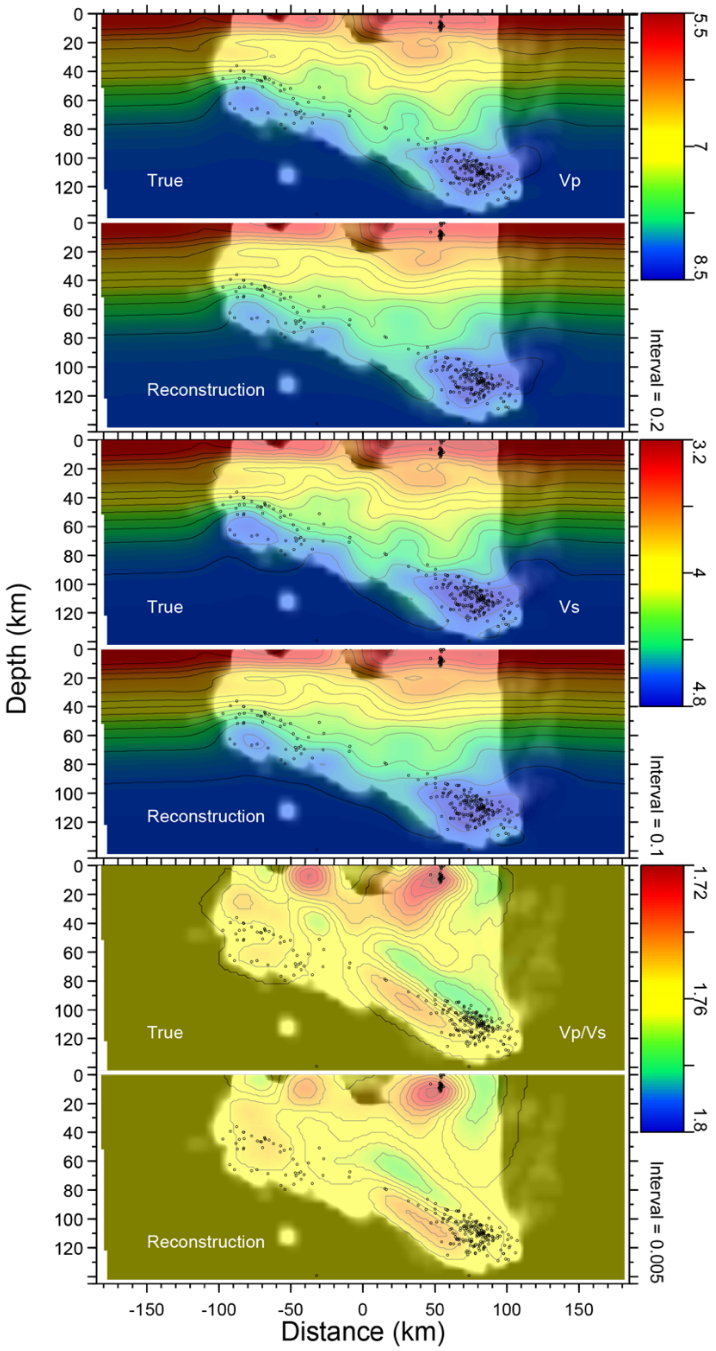

Figure S9. Results of the reconstruction tests for Vp (top two panels), Vs (middle two panels) and Vp/Vs (bottom two panels). In each case the "True" model, taken from the preferred model, is

shown at the top, and the reconstructed model directly beneath. These sections correspond to P12 in Figures S4[6]. We use the GMT software version 5.0 (https://www.generic-mapping-tools.org) for generating the map.

2.6 Data misfit

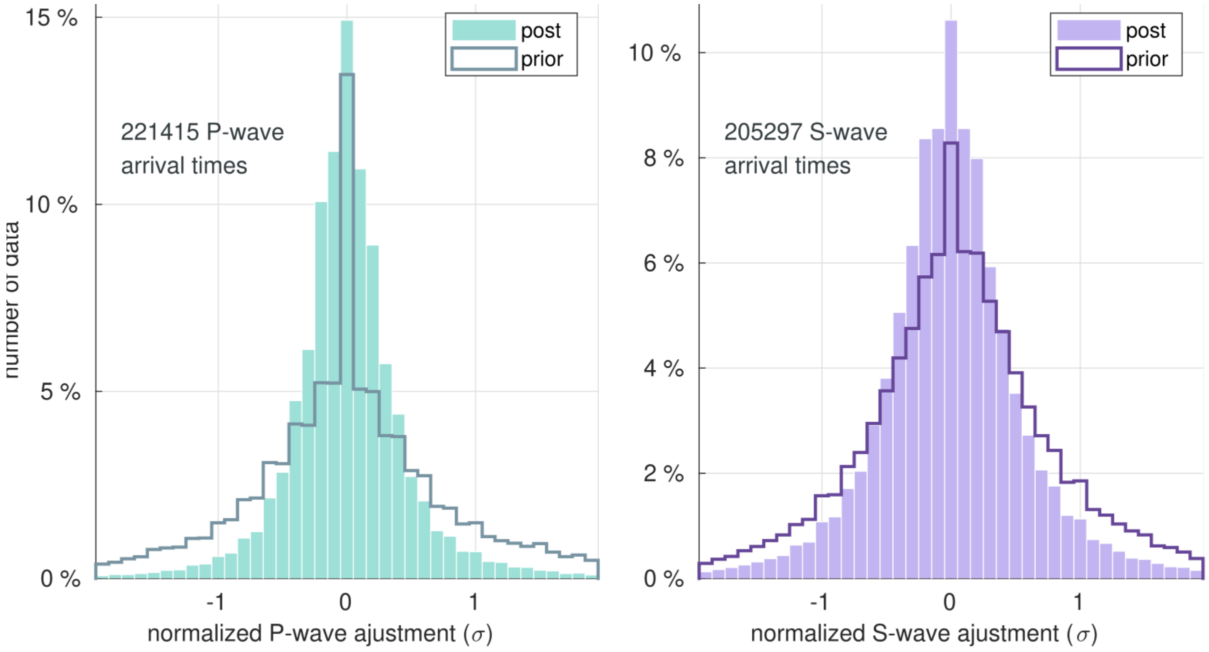

Figure S10. Arrival-times misfit before and after inversion, expressed in observational uncertainties σ. We use the GMT software version 5.0 (https://www.generic-mapping-tools.org) for generating the map.

References

1. Ślęzak, K., Díaz, D., Vargas, J. A., Cordell, D., Reyes-Cordova, F., and Segovia, M. J. Magnetotelluric image of the Chilean subduction zone in the Salar de Atacama region (23°-24° S): Insights into factors controlling the distribution of volcanic arc magmatism. *Physics. Earth. Planet. Int.*, doi: doi.org/10.1016/j.pepi.2021.106765, (2021).
2. Husen, S., Kissling, E., Flueh, E., and Asch, G. Accurate hypocenter determination in the seismogenic zone of the subducting Nazca plate in north Chile using a combined on-/offshore network, *Geophys. J. Int.*, 138, 687-701. doi.org/10.1046/j.1365-246x.1999.00893.x, (1999)
3. Beck, S., Silver, P., Drake, L., Zandt, G., Myers, S. & Wallace, T., Crustal-thickness variations in the central andes, *Geology,* **24,** 407–410. (1996)
4. Yuan, X. *et al.*, New constraints on subduction and collision processes in the central Andes from *P*-to-*S* converted seismic phases, *Nature,* **408,** 958–961. (2000)
5. McGlashan, N., Brown, L. & Kay, S., Crustal thickness in the central Andes from teleseismically recorded depth phase precursors, *Geophys. J. Int.,* **175,** 1013–1022. (2008)

6. Comte, D., Carrizo, D., Roecker, S., Ortega, F., Peyrat, S. Three Dimensional Elastic Wavespeeds in the Northern Chile Subduction Zone: Variations in Hydration in the Supra-Slab Mantle, Geophys. J. Int. 207, 1080–1105, (2016)
7. Kennett, B. L. N.; Engdahl, E. R. & Buland, R. Constraints on seismic velocities in the Earth from traveltimes, *Geophys. J. Int.*, 122, 108-124, (1995)
8. Ji, S.; Li, L.; Motra, H. B.; Wuttke, F.; Sun, S.; Michibayashi, K. & Salisbury, M. H. Poisson s ratio and auxetic properties of natural rocks, *J. Geophys. Res.: Solid Eart*h, 123, 1161-1185. doi:https://doi.org/10.1002/2017JB014606, (2018)
9. Montagner, J.-P., Regional three-dimensional structures using long- periodsurface waves, *Ann. Geophys.,* **4,** 283–291. (1986)
10. Gomberg, J.S. and Masters, T.G., Waveform modelling using locked- mode synthetic and differential seismograms: application to determination of the structure of Mexico, *Geophys. J. Int.,* **94,** 193–218. (1988)
11. Bensen, G.M., Ritzwoller, M., Barmin, M., Levshin, A., Lin, F., Moschetti, M., Shapiro, N. & Yang, Y., Processing seismic ambient noise data to obtain reliable broad-band surface wave dispersion measurements, *Geophys. J. Int.,* **169,** 1239–1260. (2007)
12. Pastén-Araya, F. Potin, B., Ruiz, S., Zerbst, L., Aden-Antoniów, F., Azúa, K., Rivera, E., Rietbrock, A., Salazar, P. and Fuenzalida, A. Seismicity in the upper plate of the Northern Chilean offshore forearc: Evidence of splay fault south of the Mejillones Peninsula, *Tectonophysics*, Volume 800, 228706, ISSN 0040-1951, https://doi.org/10.1016/j.tecto.2020.228706. (2021).
13. Tarantola, A. & Valette, B. Inverse Problems = Quest for Information. *Journal of Geophysics*, 50, 159-170, (1982)
14. Araujo, S.; Valette, B.; Potin, B. & Ruiz, M. A preliminary seismic travel time tomography beneath Ecuador from data of the national network. *J. South Am. Earth Sci.*, https://doi.org/10.1016/j.jsames.2021.103486, (2021)
15. Podvin, P. & Lecomte, I. Finite difference computation of traveltimes in very contrasted velocity models: a massively parallel approach and its associated tools. *Geophys. J. Int.*, (1991)
16. Paige, C. C. & Saunders, M. A. LSQR: An Algorithm for Sparse Linear Equations and Sparse Least Squares. ACM Transactions on *Mathematical Software*, (1982)
